# Chromosome dynamics near the sol-gel phase transition dictate the timing of remote genomic interactions

**DOI:** 10.1038/s41467-019-10628-9

**Published:** 2019-06-24

**Authors:** Nimish Khanna, Yaojun Zhang, Joseph S. Lucas, Olga K. Dudko, Cornelis Murre

**Affiliations:** 1 Division of Biological Sciences, 0377, Department of Molecular Biology, University of California, San Diego, La Jolla, CA 92093 USA; 2 Princeton Center for Theoretical Science, Princeton University, Princeton, NJ 08544 USA; 3 Department of Physics, University of California, San Diego, La Jolla, CA 92093 USA

**Keywords:** Biopolymers in vivo, Computational biophysics, Gene regulation, Epigenetics in immune cells, Chromatin structure

## Abstract

Diverse antibody repertoires are generated through remote genomic interactions involving immunoglobulin variable (V_H_), diversity (D_H_) and joining (J_H_) gene segments. How such interactions are orchestrated remains unknown. Here we develop a strategy to track V_H_-D_H_J_H_ motion in B-lymphocytes. We find that V_H_ and D_H_J_H_ segments are trapped in configurations that allow only local motion, such that spatially proximal segments remain in proximity, while spatially remote segments remain remote. Within a subset of cells, however, abrupt changes in V_H_-D_H_J_H_ motion are observed, plausibly caused by temporal alterations in chromatin configurations. Comparison of experimental and simulated data suggests that constrained motion is imposed by a network of cross-linked chromatin chains characteristic of a gel phase, yet poised near the sol phase, a solution of independent chromatin chains. These results suggest that chromosome organization near the sol-gel phase transition dictates the timing of genomic interactions to orchestrate gene expression and somatic recombination.

## Abstract (web summary)

Antibodies are generated through remote genomic interactions involving immunoglobulin variable (V_H_), diversity (D_H_) and joining (J_H_) gene segments. Here the authors develop a strategy to track V_H_-D_H_J_H_ motion in B-lymphocytes and provide evidence that chromosome organisation near the sol-gel phase transition dictates the timing of genomic interactions to orchestrate gene expression and somatic recombination.

## Introduction

It is now well established that the genome is folded hierarchically across multiple length scales. Chromosomes occupy discrete territories with no extensive intertwining^1–3^. At the scale of individual chromosomes, the genome is partitioned into transcriptionally permissive (euchromatic) and repressive (heterochromatic) compartments^3^. Within these compartments, the chromatin fiber is folded into clusters of loops, also known as topological associating domains (TADs) or loop domains^3–5^. Loop domains are anchored by CTCF proteins, which bind to convergent sequence motifs that are dispersed across the genome^5^. Cohesin, which is recruited by actively transcribed regions, moves along the chromatin in a progressive fashion extruding a loop until it reaches paired CTCF anchors^6–9^. Finally, there is now ample evidence that compartmentalization of the genome can result from phase separation of multi-molecular assemblies^10–16^.

B-cell development is initiated in the fetal liver or the adult bone marrow at the common lymphoid progenitor cell stage^17^. Common lymphoid progenitors give rise to pro-B cells in which the immunoglobulin heavy chain locus undergoes ordered V_H_-D_H_-J_H_ rearrangement^18^. D_H_-J_H_ joining occurs first followed by V_H_-D_H_J_H_ rearrangement. Once a productive V_H_-D_H_J_H_ rearrangement has occurred, pro-B cells differentiate into pre-B cells. At the pre-B cell stage, the immunoglobulin light chain loci, Igκ and Igλ, recombine to generate V_κ_J_κ_ or V_λ_J_λ_ joins. Upon formation of a productive light chain locus rearrangement, pre-B cells differentiate into immature-B cells that express an antibody. In the absence of auto-reactivity immature-B cells exit the bone marrow to migrate to the peripheral lymphoid organs^19^.

**Fig. 1:**
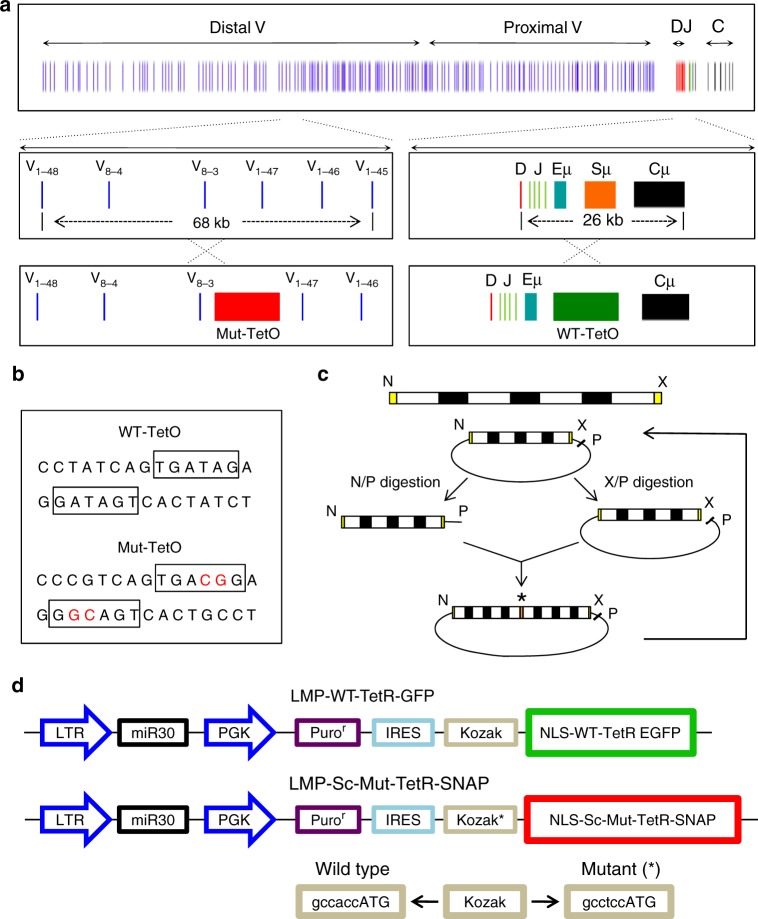
A strategy for tracking V_H_-D_H_J_H_ motion in live B cells. **a** Schematic diagram of the Igh locus showing V_H_, D_H_, J_H_, C_H_ gene segments, intronic enhancer (Eμ), and switch region repeats (Sμ). **b** DNA sequences of wild-type and mutant TET operator binding sites. Nucleotides for which the mutant binding sites differ from that of WT-TET binding sites are indicated in red. **c** Tandem arrays containing 360 copies of the MUT-TET binding site were generated by sequential cloning and expansion as described previously^47^. Each template contained four MUT-TET binding sites (white boxes). Black boxes (N10) refer to a 10 bp oligonucleotide composed of random DNA sequences. N refers to *Nhe*1, X to *Xba*1, and P refers to *Pst*1. Asterisk indicates the junction of *Nhe*1-*Xba*1 ligated DNA segments. **d** Construction of retroviral vectors to optimize the signal to noise ratio in pro-B cells transduced with virus expressing wild-type TET-EGFP and MUT-TET SNAP Tag

Antigen receptor locus rearrangement is initiated by two endonucleases, RAG1 and RAG2, which generate double-stranded DNA breaks in the recombination signal sequences flanking the V_H_, D_H_, and J_H_ segments^20^. The variable regions are segregated into two distinct clusters, the distal and proximal V_H_ regions, that collectively span ~2.7 Mb (Fig. 1). Located downstream of the constant regions (C_H_) is a cluster of CTCF sites known as the Igh 3′ regulatory region, or the Igh super-anchor^21,22^. V_H_ regions are frequently flanked by CTCF binding sites that are positioned in convergent orientation towards the super-anchor^21,22^. Two CTCF binding sites separate the V_H_ from the D_H_J_H_ elements to suppress premature and aberrant V_H_-D_H_ rearrangement^22–24^.

The Igh locus is characterized by a large spectrum of epigenetic modifications distributed across the V_H_ and D_H_J_H_ regions^25^. A subset of these modifications is deposited in a developmental stage-specific fashion and correlate with the onset of D_H_-J_H_ rearrangement^26,27^. The deposition of these marks is controlled, at least in part, by an intronic enhancer located immediately downstream of the D_H_J_H_ region^25^. V_H_-D_H_J_H_ rearrangement is associated with the deposition of epigenetic marks across the V_H_ regions^27,28^. The deposition of epigenetic marks across the Igh locus is controlled by a subset of transcriptional regulators, including E2A, EBF1, and PAX5^28–30^. Prominent among these are the E-proteins^31^. Specifically, the E2A and HEB proteins play key roles in orchestrating antigen receptor locus assembly^32–35^. They instruct the assembly of an elaborate network of crosslinks across antigen receptor, loci^36^. The E2A-proteins recruit the acetyl transferase P300 and the chromatin remodeler BRG1 to a spectrum of binding sites that span the Igh and Igκ chromatin regions^37–39^. Once recruited to E2A-bound sites, P300 acetylates lysine residues on the histone tails of H3 and H4^40^. Acetylated H3 and H4 residues, in turn, recruit the chromatin remodeler BRD4 to promote crosslinking and phase separation across the chromatin fiber^11,12^. In activated mature-B cells the E2A proteins assemble into droplets^41^.

Recent studies using a single-color labeling strategy demonstrated that, governed by a viscoelastic environment, V_H_ and D_H_J_H_ elements undergo anomalous diffusion, bouncing back and forth in a spring-like fashion^42–44^. However, since only a single element (V_H_ or D_H_J_H_) was fluorescently marked, these studies did not permit the visualization of V_H_-D_H_J_H_ motion.

Here, we develop a two-color labeling strategy, which allows us to visualize and characterize V_H_-D_H_J_H_ motion. Specifically, we generate tandem arrays of wild-type TET-operator and mutant TET-operator binding sites to separately mark the V_H_ and the D_H_J_H_ elements. We find that in the majority of cells the Igh locus adopted a wide spectrum of configurations that are dynamic enough to allow local motion yet stable enough to provide a strong long-term confinement. Notably, however, a subset of cells display increased V_H_-D_H_J_H_ motion plausibly caused by temporal changes in chromatin configurations. To determine the mechanistic origin of this confined motion, we perform Molecular Dynamics simulations of a hierarchy of polymer models. The comparison of the simulated and experimental data reveals that the Igh loop domains are organized as gel droplets poised near the sol–gel phase transition. We propose that the proximity of the locus to the sol–gel phase boundary provides a tradeoff between stability and adaptability characteristic of the gel and sol phases, respectively. The ordered nature of the crosslinked network, formed by the locus and characteristic of a gel, enables ordered Igh locus rearrangement by facilitating and insulating V_H_-D_H_J_H_ and D_H_-J_H_ encounters. At the same time, the disordered nature of a solution of free chains, characteristic of a sol, permits randomness in V_H_-D_H_J_H_ encounters and rapid locus re-assembly. 

## Results

### An approach to monitor genomic interactions in live cells

In previous studies we tracked either V_H_ or D_H_J_H_ motion in live pro-B cells using tandem arrays of TET-operator binding sites^42^. We next aimed to monitor V_H_-D_H_J_H_ encounters directly in live cells using a dual labeling approach (Fig. 1a). Attempts to insert tandem arrays of LAC operators in conjunction with TET-operator binding sites failed because tandem arrays of LAC operator binding sites were unstable in expanding pro-B cell cultures. We therefore developed a strategy to simultaneously track the motion of paired genomic elements. Specifically, we used wild-type and mutant TET-repressor binding sites associated with unique DNA binding specificities^45,46^. From these, we selected a mutant TET repressor (4C5G) carrying two amino acid substitutions (E37A and P39K) in the TET-DNA binding domain (Fig. 1b). Tandem arrays containing 360 copies of the mutant TET 4C5G binding site (CCGTCAGTGACGG) were constructed essentially as described previously (Fig. 1c)^47^.

In principle, two distinct repressor modules should permit the simultaneous tracking of two genomic regions. However, heterodimers involving wild-type TET and mutant TET would readily be assembled interfering with their ability to selectively bind their cognate homo-dimeric binding sites. To overcome this obstacle, we converted the MUT-TET repressor dimer into a single chain using a 29 amino acid linker. The single chain (sc) TET-repressor binds to its cognate binding site as a monomer^45^. To generate two differentially labeled fusion proteins, the wild-type TET-repressor was fused to GFP while the mutant (sc) TET (4C5G) repressor was tagged with a polypeptide (SNAP-TAG) that binds cell-permeable fluorescent dyes (Fig. 1d). To boost expression of the wild-type and mutant TET-repressors, both proteins were codon-optimized. Finally, to optimize the signal-to-background fluorescence ratio, the TET-SNAP-tag repressor was placed under control of a mutant Kozak sequence (GCCTCCATG) to dampen translation and reduce background fluorescence (Fig. 1d).

### Tracking V_H_-D_H_J_H_ motion in live B-lymphocytes

**Fig. 2:**
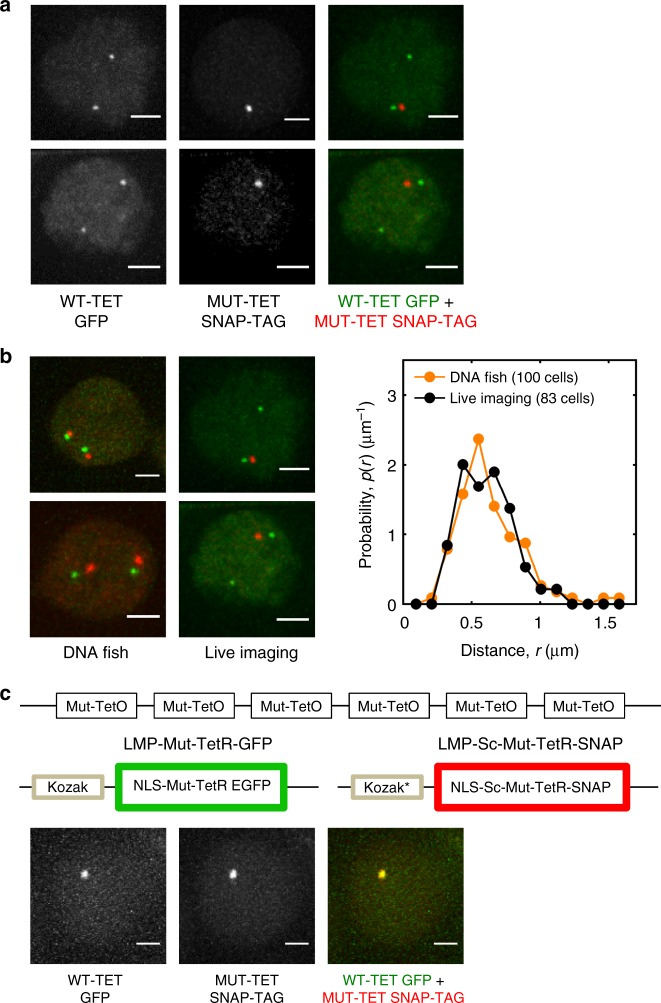
Tracking V_H_-D_H_J_H_ motion in pro-B cells. **a** Live imaging of pro-B cells harboring D_H_J_H_ WT-TET and V_H_ MUT-TET alleles. Panels indicate pro-B cells carrying D_H_J_H_ WT-TET and V_H_ MUT-TET alleles transduced with WT-TET GFP and MUT-TET SNAP-TAG. Left panels display pro-B cells transduced with virus expressing WT-GEP. Middle panels show pro-B cells transduced with virus expressing MUT-TET SNAP-TAG. Right panels show pro-B cells transduced with WT-GFP and MUT-TET SNAP-TAG. Scale bar corresponds to 2 μm. **b** Left panels show pro-B cell lines harboring tandem arrays of WT-TET GFP and MUT-TET SNAP-TAG analyzed using 3D-FISH. Fluorescently labeled V_H_ and D_H_J_H_ probes were used for visualization. Middle panels show fluorescent foci obtained from live-cell imaging. Right panel shows the probability distributions of spatial distances separating the V_H_ and D_H_J_H_ elements obtained from live pro-B cell imaging (black dots) compared to those obtained from 3D-FISH measurements (orange dots). Numbers of cells that were analyzed is indicated. **c** Experimental strategy is displayed to determine localization error. Localization error was determined by measuring the spatial distances separating red and green fluorescent signals. Fifty cells were analyzed. Scale bars represents 10 μm. Source data are provided as a Source Data File

In previous studies, we generated mice that carried tandem arrays of WT-TET operator binding sites in either the V_H_ or D_H_J_H_ regions^43^. From these mice we derived immortalized RAG-deficient pro-B cell lines. Briefly, RAG2-deficient WT-TET D_H_J_H_ pro-B cells were isolated, grown in the presence of IL7 and SCF and transduced with BCR-ABL virus. This approach yielded BCR-ABL-transformed pro-B cell lines that carried a tandem array of TET-operator binding sites adjacent to the D_H_J_H_ region (Fig. 1a). Next, using CAS9-CRISPR engineering a tandem array of mutant TET repressor binding sites was inserted into the V_H_ 8-3 region (Supplementary Fig. 1). Cells that harbored the array of MUT-TET binding sites in the V_H_ 8-3 region were expanded and transduced with virus expressing WT-TET GFP and MUT-TET SNAP-TAG. Two days post viral transduction the cells were incubated with the cell-permeable dye TMR-STAR to label MUT-TET bound sites and imaged using the OMX microscope platform. Cells transduced with virus expressing WT-GFP revealed two fluorescent foci consistent with WT-TET binding near the D_H_J_H_ region at both alleles (Fig. 2a; left panels). Cells transduced with virus expressing MUT-TET SNAP TAG revealed a single fluorescent center consistent with the insertion of MUT-TET binding sites on one allele (Fig. 2a, middle panels). Cells transduced with virus expressing WT-TET GFP and MUT-TET SNAP TAG showed one allele marked for both the V_H_ and D_H_J_H_ regions and another allele marked with only the D_H_J_H_ region (Fig. 2a, right panels). Transduced V_H_ MUT-TET; D_H_J_H_ WT-TET pro-B cells were imaged every 2 s for 400 s or every 40 s for 4800 s. We found that, depending on the efficiency of viral transduction, the V_H_ and D_H_J_H_ region were marked by both red and green fluorescence in 1–10% of the transduced pro-B cell population.

To validate this approach, we compared the distribution of the spatial distances separating the V_H_ and D_H_J_H_ elements obtained from live-cell imaging to those obtained from three- dimensional (3D)-FISH measurements (Fig. 2b). We found that both methods yielded similar distributions, consistent with the insertion of tandem arrays of WT-TET and MUT-TET binding sites into the V_H_ and D_H_J_H_ regions (Fig. 2b, right panel). To measure localization error, pro-B cells harboring tandem arrays of MUT-TET binding sites were transduced with virus expressing MUT-TET GFP (green fluorescence) and virus expressing MUT-TET SNAP-TAG (red fluorescence) (Fig. 2c). Cells that showed overlapping green and red fluorescent foci were imaged every 2 s for 400 s. The mean-squared displacement was ~0.02 µm^2^, i.e., an average localization error of 22 nm for the *x*- and *y*-coordinates and 95 nm for the *z*-coordinate for each marker. To extract the intrinsic V_H_-D_H_J_H_ genomic motion from the observed motion, rotational, and translational nuclear motion, as well as localization error were eliminated using two independent procedures (Supplementary Fig. 2). Neither procedure involved adjustable parameters and both yielded very similar outcomes (Supplementary Fig. 2). These results show that the motion of paired coding or regulatory elements can be tracked with relatively high accuracy using a combination of wild-type and mutant TET operator binding sites.

### V_H_-D_H_J_H_ trajectories revealed highly constrained motion

**Fig. 3:**
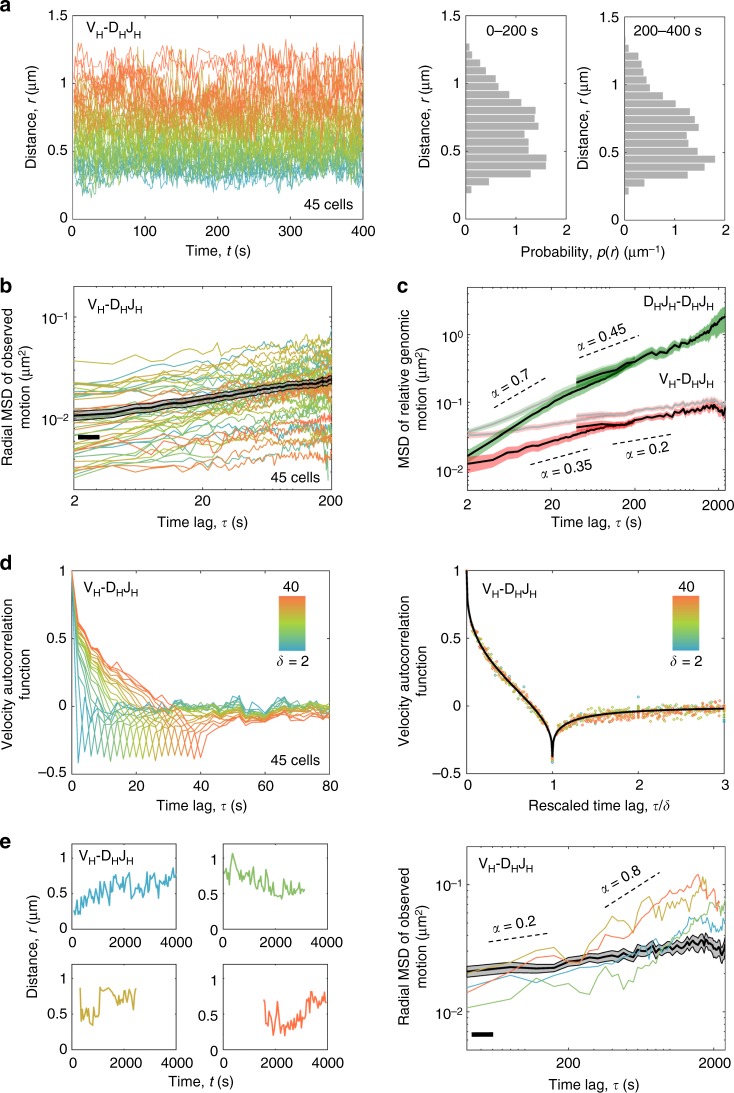
V_H_-D_H_J_H_ motion in B-cell progenitors is extremely confined. **a** The spatial distances separating V_H_ and D_H_J_H_ elements for the majority of cells fluctuated around their respective average values that remained nearly constant over time. Color-coding the distance trajectories according to their mean values reveals a “demixing” effect. Right panel shows the corresponding probability distribution of V_H_-D_H_J_H_ distances. **b** The time-averaged radial MSD (colored lines) and the time-and-ensemble-averaged radial MSD (black line) for V_H_-D_H_J_H_ motion. **c** Time-and-ensemble-averaged MSD of relative genomic motion for inter-chromosomal D_H_J_H_-D_H_J_H_ pair (green) and intra-chromosomal V_H_-D_H_J_H_ pair (red) before (pale lines) and after (bright lines) measurement-error correction. **d** Velocity autocorrelation functions for the V_H_-D_H_J_H_ motion exhibit anti-correlations indicative of a push-back from the environment; the value at the dip approaches the theoretical limit (−0.5) of an extreme confinement. The collapse of the velocity autocorrelation function curves upon rescaling of the time axis (right panel) indicates self-similarity of motion. Source data are provided as a Source Data file. **e** Left panel shows the V_H_-D_H_J_H_ trajectories that, unlike those in **a**, exhibited substantial changes in their mean values over time. Right panel shows the radial MSD curves (colored lines) for the individual cells that displayed abrupt changes in V_H_-D_H_J_H_ motion and, for comparison, the time-and-ensemble-averaged radial MSD (black line) for V_H_-D_H_J_H_ motion in the majority of cells. Scaling exponents (*α*) are indicated. Source data are provided as a Source Data File

Individual trajectories of the spatial distances separating the labeled V_H_ and D_H_J_H_ regions revealed that, for the majority of cells (90%), V_H_-DJ_H_ motion was highly constrained: the V_H_ and D_H_J_H_ segments that were initially in spatial proximity remained in proximity, whereas the segments that were initially spatially remote explored their immediate neighborhood while remaining remote (Fig. 3a and Supplementary Fig. 3). The corresponding probability distribution of V_H_-D_H_J_H_ distances that was generated from these trajectories covered a broad range of distances (0.2–1.2 μm) suggesting that, across the population of cells, the locus adopts a wide spectrum of chromatin configurations (Fig. 3a, right panel). Notably, the distribution exhibited bimodality (*p*-value of 0.003 returned by Hartigan’s Dip Test indicated that bimodality was statistically significant) that persisted for at least 400 s, indicating that at least two dominant types of chromatin configurations were associated with Igh locus topology. Color-coding the temporal trajectories of V_H_-D_H_J_H_ spatial distances according to their mean values revealed an intriguing “demixing” effect: the distances within the individual V_H_-D_H_J_H_ pairs fluctuated around their respective mean values that remained nearly constant, together resulting in a “rainbow” pattern that persisted for at least an hour (Fig. 3a, left panel; Supplementary Fig. 3). These data indicate that the Igh locus adopts a wide spectrum of configurations that are sufficiently dynamic to allow local motion yet stable enough to provide the long-term confinement that either co-localizes V_H_ and D_H_J_H_ segments or isolates them from each other.

The properties of V_H_-D_H_J_H_ motion averaged across the trajectories were examined by computing the mean-squared displacements (MSD) and the velocity autocorrelation functions (Fig. 3b–d). The velocity autocorrelation function reveals the degree of correlation between the average velocities of a pair of segments at two instants separated by the time interval *τ* (the velocity itself is the average displacement over the time interval *δ*). MSD and velocity autocorrelation function were calculated using pairwise V_H_-D_H_J_H_ and D_H_J_H_-D_H_J_H_ distance trajectories, which eliminated the effect of nuclear motion. Time-averaged as well as time-and-ensemble-averaged MSD exhibited a subdiffusive scaling exponent (*α* < 1) both for intra-chromosomal V_H_-D_H_J_H_ and inter-chromosomal D_H_J_H_-D_H_J_H_ motion (Fig. 3b, c and Supplementary Fig. 4; Supplementary Table 1). Notably, V_H_-D_H_J_H_ motion was found to be extremely subdiffusive, characterized by a scaling exponent *α* that decreased from 0.35 at short time scales to 0.2 at long time scales (Fig. 3c and Supplementary Table 1). The velocity autocorrelation functions exhibited negative correlations indicative of a push-back from the environment (Fig. 3d). For intra-chromosomal motion, the value of the velocity autocorrelation function at the dip approached the theoretical limit (−0.5) of an extreme confinement (Fig. 3d). The collapse of the velocity autocorrelation curves upon rescaling of the time axis indicated that the motion was self-similar, i.e., exhibited similar patterns at different spatial and temporal scales (Fig. 3d, right panel). Thus, the trajectories from the vast majority of imaged cells displayed substantial spatial confinement imposed on the V_H_-D_H_J_H_ motion. We note, however, that a significant fraction (10%) of cells revealed trajectories that appeared less constrained and displayed abrupt changes in the scaling exponent ranging from 0.2 to 0.8 during the course of imaging (Fig. 3e). Taken together, these measurements indicate that, while in the majority of pro-B cells V_H_-D_H_J_H_ motion was strongly subdiffusive and predominantly governed by a viscoelastic and spatially confining neighborhood, a subset of pro-B cells revealed V_H_-D_H_J_H_ trajectories operating in a significantly less constrained environment.

### Chromatin loops provide global confinement

**Fig. 4:**
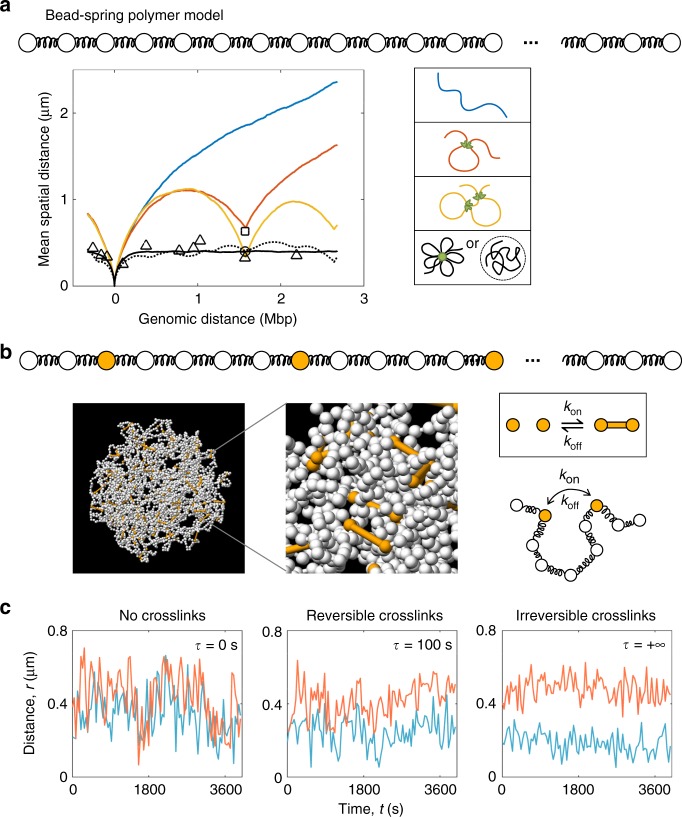
Molecular dynamics simulations capture Igh locus structure and dynamics. **a** The mean spatial separation as a function of the genomic distance between the D_H_J_H_ and the markers that span the V_H_ regions are indicated. Triangles represent the mean spatial separation between the marked regions and the D_H_J_H_ elements as measured previously by 3D-FISH^49^. Note that the plotted average values were obtained for > 100 RAG-deficient pro-B cells for all shown data points^49^. The square and circle represent the mean spatial separation between the D_H_J_H_ region and the V_H_3 regions cultured in the absence (*n* = 121) or presence (*n* = 45) of STI-571, respectively. Colored lines show spatial separation as a function of genomic distance for the IgH locus modeled as an unstructured chromatin chain (blue line), a single loop configuration (red line) or a two-loop configuration (orange line). Right panel indicates the different configurations that were analyzed. **b** A snapshot extracted from MD simulations of the bead-spring polymer model with cross-linkable sites spanning the Igh locus. **c** Two representative trajectories of V_H_-D_H_J_H_ spatial distances from simulations (red and blue lines) demonstrate how the insertion of crosslinks across the locus in the simulations reproduces the experimentally observed “demixing” of the trajectories. Source data is provided as a Source Data File

To explore the mechanistic origin of the viscoelasticity and confinement that govern V_H_-D_H_J_H_ motion, we modeled the chromatin fiber as a bead-spring polymer using molecular dynamics (MD) simulations (Fig. 4)^48^. Given the large number of molecular components and an extensive parameter space that could be incorporated in the simulations, we constrained the model by utilizing multiple independent sets of experimental data. As a first step, we sought to identify the minimal model that could reproduce the structural properties of the Igh locus, namely the plateau in the V_H_-D_H_J_H_ spatial distances as a function of the genomic distance as observed in 3D-FISH^49,50^. As a second step, the resulting minimal model was refined such that it could also reproduce the dynamic properties of the locus, namely the near-constant average spatial distances separating the V_H_ from the D_H_J_H_ regions and the strongly subdiffusive scaling exponent associated with V_H_-D_H_J_H_ motion.

To capture the structural properties of the Igh locus, we simulated a hierarchy of worm-like chain polymer models reflecting chromatin configurations of increasing complexity: an unstructured chromatin chain, a single loop configuration, a two-loop configuration, and a chain enclosed in a confinement that mimicked a multiple-loop configuration (Fig. 4a and Supplementary Methods). As expected, the unstructured chain model failed to generate a plateau-like relationship between spatial and genomic V_H_-D_H_J_H_ distances (Fig. 4a). A spatial confinement enclosing the chain was necessary to reproduce the plateau observed in 3D-FISH (Fig. 4a, solid black line)^49,50^. Notably, the average over a large number of explicit multiple-loop configurations in the absence of confinement also reproduced the plateau in the spatial vs. genomic distances (Fig. 4a, dotted black line). These results indicate that chromatin loops can act as a primary source of large-scale confinement to provide an equal playing field for the V_H_ region repertoire independent of genomic separation.

### Immunoglobulin heavy chain locus in a weak-gel state

While the model of the Igh locus organized as loops reproduced the structural properties of the locus, it yielded spatial distances that fluctuated considerably, resulting in intersecting trajectories (Fig. 4c, left panel). These trajectories were inconsistent with the “demixed” V_H_-D_H_J_H_ trajectories observed in live-cell imaging, in which the spatial distances only exhibited local fluctuations (Fig. 3a and Supplementary Fig. 3). This discrepancy pointed to yet another layer of spatial constraint, which confined the motion locally. A plausible mechanism of the local confinement is cross-bridging of the chromatin fiber, analogous to the mechanism recently proposed for super-enhancers whereby crosslinks induce phase-separated condensates^13^. To examine a potential role for crosslinks as the source of local confinement, we introduced 5% cross-linkable sites that could undergo pairwise binding and unbinding with tunable kinetics (Fig. 4b and Supplementary Methods). Notably, gradually increasing the cross-link residence time resulted in a progressive suppression of the fluctuations in the spatial distances separating the paired genomic elements, ultimately recovering the effect of “demixing” of the trajectories (Fig. 4c).

**Fig. 5:**
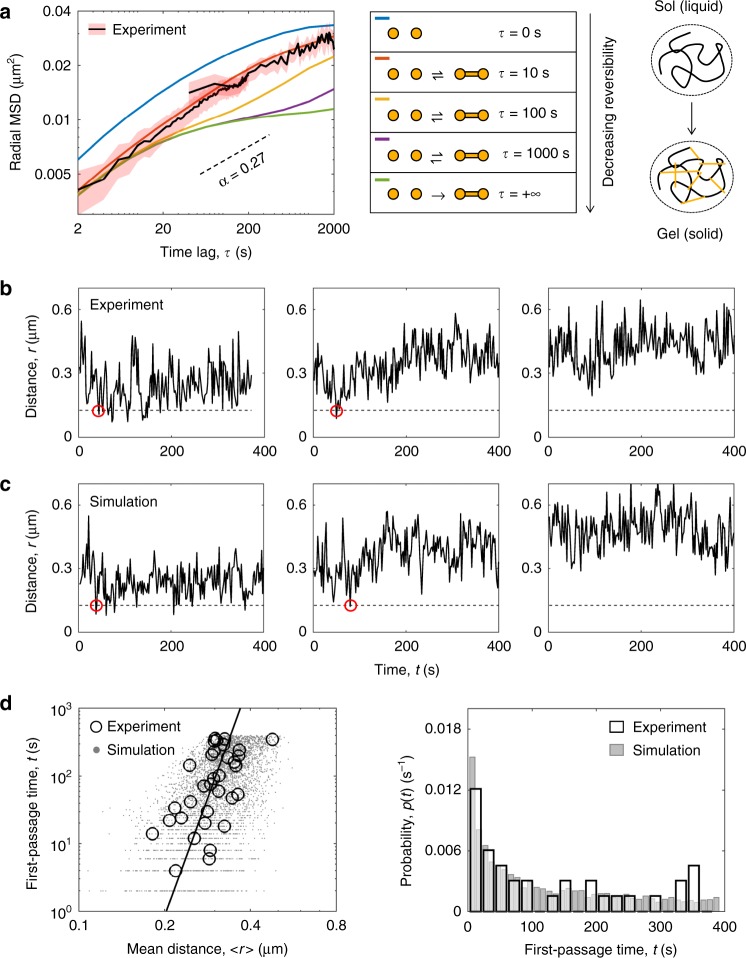
A weak-gel model reproduces V_H_-D_H_J_H_ first-encounter times. **a** Tuning the lifetime of crosslinks results in a systematic change in the slope (*α*) of the MSD. Short-lived bonds (lifetime ~10 s) yield the best agreement between MSD derived from experiment and simulations. **b** Representative trajectories of the spatial distance within individual V_H_-D_H_J_H_ pairs in STI-571 treated cells (*n* = 121). Red circles indicate potential first encounters. **c** Representative trajectories of V_H_-D_H_J_H_ spatial distances generated from the MD simulations of the Igh locus when modeled as a weak gel. **d** First encounter times as a function of mean spatial separation from experimental and simulated V_H_-D_H_J_H_ trajectories (left panel), and the corresponding probability distributions of experimentally derived and simulated first-encounter times (right panel). Source data is provided as a Source Data File

A network of crosslinked polymer chains and a solution of free (un-crosslinked) chains represent two distinct physical phases: a gel phase (a solid phase) and a sol phase (a liquid phase), respectively^51,52^. Given that the ordered nature of the solid phase and the fluidity of the liquid phase can both be advantageous from a biological prospective, we asked: to what extent are the properties of Igh locus solid-like as opposed to liquid-like? In other words, how deep in the gel phase is the locus positioned on a phase diagram? The tuning of the bond lifetime of the simulated crosslinks from irreversible bonds (strong gel) to short-lived bonds (weak-gel) and further to the absence of crosslinking (sol) resulted in a systematic change in the slope (*α*) of the MSD (Fig. 5a). The bond lifetime on the order of 10 s was found to yield the best agreement between simulated and experimentally measured MSD. The short-lived nature of the crosslinks indicates that the state of the locus corresponds to a weak gel poised close to the boundary of gel and sol phases (Fig. 5a). These results suggest that a global confinement imposed by chromatin loops and a local confinement imposed by the cooperative action of short-lived crosslinks together constitute the mechanistic origin of the observed strongly subdiffusive V_H_-D_H_J_H_ motion.

### V_H_-D_H_J_H_ encounter times in a weak-gel state

The two-color imaging strategy enabled a direct estimation of the first-passage times (FPTs), or the waiting times of the first V_H_-D_H_J_H_ encounters (Fig. 5b and Supplementary Fig. 6). We set a threshold value for the distance at which the segments can be considered interacting by combining the value of 30 nm for the true interaction distance and the contribution from the localization error. A dip in the distance below the threshold indicates a potential encounter, and the corresponding time provides an estimate for the FPT. In order to acquire a larger fraction of cells in which V_H_ and D_H_J_H_ segments were co-localized within close spatial proximity, we generated a pool of pro-B cells cultured in the presence of STI-571. STI-571 treated BCR-ABL-transformed cells undergo large-scale nuclear contraction through mechanisms yet to be determined. In the sub-population of the cells in which V_H_ and D_H_J_H_ segments were spatially remote at the start of imaging (initial separation > 0.55 µm), no potential encounters were detected throughout the imaging period. In contrast, in the sub-population of cells in which V_H_ and D_H_J_H_ segments were spatially localized within relatively close spatial proximity at the start of imaging (<0.55 µm), the potential encounters occurred within minutes in >40% of these cells (Fig. 5b). To examine whether the weak-gel model could reproduce experimentally observed first-encounter times, we generated the trajectories of V_H_-D_H_J_H_ distances from Molecular Dynamics simulations of a globally confined, weakly crosslinked chain. The range of first-encounter times extracted from the simulated trajectories was in quantitative agreement with the range observed experimentally (Fig. 5b, c). To investigate the link between temporal and spatial aspects of the genomic encounters, we examined both the measured and simulated FPTs as a function of the V_H_-D_H_J_H_ mean spatial distance, which sets the length scale (*R*) of this first-passage-time problem (Supplementary Methods). The experimental and simulated data showed good quantitative agreement (Fig. 5d). Furthermore, on a log-log plot, both types of data resulted in the best fit by a line with a slope of 2/*α* (Fig. 5d and Supplementary Fig. 7), in agreement with the predicted scaling MFPT ~ *R*^2/*α*^^43,44^. We conclude that the model of the Igh locus poised near the sol/gel phase boundary generates the time scales of the V_H_-D_H_J_H_ encounters that are in quantitative agreement with those measured experimentally.

## Discussion

It is now established that the Igh locus is organized as loops^22–25,49,50^. However, our understanding of the mechanisms that govern the dynamics of V(D)J rearrangement remains rudimentary. The approach developed here enabled us to simultaneously track V_H_ and D_H_J_H_ motion and to visualize V_H_-D_H_J_H_ encounters. The resulting findings highlight the link between the spatial and temporal aspects of Igh locus organization. The interplay between the spatial confinement imposed by loop domains and crosslinks on the one hand and the inherently subdiffusive dynamics of the chromatin fiber on the other hand can either facilitate or hinder genomic interactions. Subdiffusive motion, in comparison to normal diffusion, has its advantages and disadvantages in mediating V_H_-D_H_J_H_ encounters: a sub-diffuser searches more efficiently over short distances but less efficiently over long distances. V_H_ and D_H_J_H_ segments are brought together through looping and their spatial proximity is further reinforced by crosslinks. This amplifies the efficiency of their subdiffusive search over short distances, thereby facilitating their encounters. Conversely, V_H_ and D_H_J_H_ gene segments that end up in different loops, which are further stabilized by crosslinks, become isolated from each other. This augments the inefficiency of their subdiffusive search over long distances, further hindering their encounters.

**Fig. 6:**
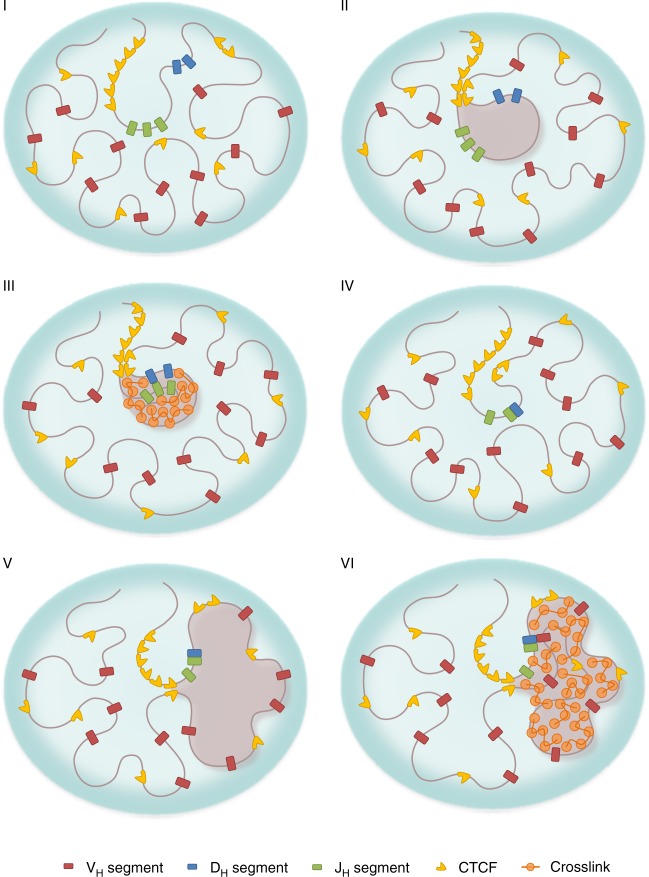
The role of gelation in orchestrating ordered Igh locus rearrangement. (I) Igh locus harboring D_H_, J_H_, and V_H_ regions in a germ-line configuration, in the absence of loop domains or crosslinks. (II) A chromatin loop domain containing D_H_ and J_H_ regions is formed, anchored by the CTCF sites flanking the D_H_ elements and the CTCF sites in the super-anchor. (III) Weak crosslinks are established across the loop domain containing D_H_ and J_H_ regions. The resulting gel droplet, which is phase-separated from the rest of the locus, facilitates encounters between D_H_ and J_H_ elements and hinders encounters with V_H_ elements. (IV) The CTCF sites are inactivated and crosslinks are dissolved; the mobility of D_H_J_H_ segment is increased. (V) Chromatin loops containing D_H_J_H_ and V_H_ regions are formed, anchored by the CTCF sites within the V_H_-cluster and the CTCF sites in the super-anchor. (VI) Crosslinks across a loop are established, resulting in a de novo gel droplet to facilitate V_H_-D_H_J_H_ encounters

Our study suggested that the structural and dynamic properties of the Igh locus are consistent with those of a weak gel, or a network of chromatin chains and associated factors that are bridged via short-lived crosslinks. We conclude that, in the phase space, the Igh locus is positioned within the gel phase (and thus it is a solid) but close to the sol/gel (or liquid/solid) phase boundary^53^. Such an organization is akin to a molded pudding with barely enough gelatin to hold its form. The lifetime of crosslinks in the cell is likely regulated, and thus represents a dynamic quantity. Our findings therefore suggest that the gelation of chromatin is a biological process that is finely tuned with respect to a dynamic variable—the strength of the crosslinks. Proximity to the liquid/solid phase boundary allows the loop domains within the Igh locus to rapidly switch their chromatin state. Tuning the chromatin properties further into the solid phase (gel) promotes the formation of phase-separated gel droplets in which encounters are facilitated between gene segments that are trapped within the same droplet and hindered otherwise (Fig. 6). Conversely, tuning the chromatin state closer to the liquid phase (sol) allows the existing gel droplets to dissolve so that de novo gel droplets can readily form. Proximity to the sol/gel phase transition thus enables both a rapid and ordered re-assembly of the Igh locus (Fig. 6).

While the proposed weak-gel model was demonstrated here to be consistent both with structural and dynamic experimental data, additional and/or other mechanisms may contribute to or underpin V_H_-D_H_J_H_ motion. To unambiguously demonstrate the effect of crosslinks on the biophysical state of the Igh locus and its influence on V_H_-D_H_J_H_ motion and somatic recombination, the consequences of interfering with these crosslinks need to be examined. What is the nature of the putative molecular components that promote crosslinking? How are these components deposited and regulated? Previous studies indicated an elaborate network of intra-chromosomal interactions in pro-B cells that were CTCF-independent and associated with E2A, PU.1, FOXO1, and PAX5 occupancy^36^. E2A occupancy is particularly intriguing since the E2A proteins regulate the assembly of antigen receptor loci^31^. E2A proteins also assemble into droplets during developmental progression akin to other transcriptional regulators^41^. Genetic and biochemical evidence have demonstrated that they act mechanistically by recruitment of P300 and BRG1 to E2A-bound sites^36–39^. A direct role for BRG1 in gelation remains to be determined but P300 is known to acetylate lysine residues located on H3 and H4 tails, which in turn, recruits BRD4^40^. Additionally, the E2A proteins themselves contain transactivation domains that are largely disordered and may assemble into gel droplets as described for other transcriptional regulators^13^.

While the majority of cells displayed strongly subdiffusive V_H_-D_H_J_H_ motion, a small fraction showed abrupt alterations in diffusive behavior that were associated with a substantial increase in the scaling exponent *α*. We propose that the observed sudden changes in the trajectories adopted by V_H_ and D_H_J_H_ elements were caused by temporal changes in chromatin configurations. Such abrupt changes in trajectories may involve loop extrusion, DNA replication or nuclear deformations. Loop extrusion is a particularly intriguing possibility since it would reshuffle V_H_ regions across the Igh locus to allow other V_H_ regions to access the recombination center in an otherwise highly spatially constrained environment.

Our findings demonstrate how the spatial and temporal aspects of genome organization intersect to regulate antigen receptor assembly. Specifically, since V_H_-D_H_J_H_ motion is strongly subdiffusive, only those coding elements that are located within relatively close spatial proximity will have a chance to encounter each other. This raises the question: how is a diverse antigen receptor repertoire established? We propose the following mechanism. First, loop extrusion would generate a D_H_-J_H_ loop domain. Once a D_H_-J_H_ loop domain has been assembled, transcription factor-induced crosslinks would promote the formation of a gel droplet; the gel droplet, in turn, would facilitate D_H_-J_H_ encounters within the loop domain and simultaneously function as an insulator to prevent V_H_-D_H_ rearrangement prior to the formation of D_H_J_H_ joints (Fig. 6). Upon the formation of a D_H_J_H_ rearrangement, transcription factor activity would decline and epigenetic marks would be erased, dissolving the droplet. The disassembly of the D_H_J_H_ droplet would be followed by the formation, through loop extrusion, of a de novo loop domain containing V_H_ and D_H_J_H_ elements^54^. Upon the assembly of the V_H_-D_H_J_H_ loop domain, transcription factors such as E2A, EBF1, and PAX5, would instruct P300 to acetylate H3 and H4 residues across the V_H_ region, promoting the formation of a gel droplet in which V_H_ regions positioned in spatial proximity to the D_H_J_H_ joint would be enabled to undergo V_H_-D_H_J_H_ rearrangement. Thus, rather than the entire spectrum of V_H_ gene segments continuously attempting to make a connection with D_H_J_H_ elements, only a selected few that are positioned nearby will have a chance to interact.

Transcriptional regulators have the ability to both establish and erase acetylation and hence have the ability to rapidly assemble and dissociate gel droplets^31^. These properties allow for a rapid response upon formation of a productive rearrangement to suppress continued antigen receptor assembly and enforce allelic exclusion as demonstrated for the E2A proteins^34^. Thus, we suggest that allelic exclusion is enforced by transcription factor-induced dissociation of gel droplets to suppress continued antigen receptor assembly. In sum, we propose that gelation within chromatin loop domains, induced by transcriptional regulators, epigenetic modifications and chromatin remodelers, facilitates and hinders V_H_-D_H_J_H_ and D_H_-J_H_ encounters thereby enforcing stepwise antigen receptor locus rearrangement and the allelic exclusion process. The proximity of the Igh locus to the sol/gel boundary in the phase space, and the resulting propensity to switch between solid and liquid states, offers a tradeoff between stability and responsiveness that underpins the mechanism that orchestrates gene expression and antigen receptor assembly.

## Methods

### Insertion of MUT-TET repressor binding sites

Tandem arrays containing 360 copies of the MUT-TET binding site (TCCCCGTCAGTGACGGAGA) were generated by sequential cloning and expansion^47^. The tandem array was inserted into a template that also contained a blasticidin selectable marker and two homology arms positioned immediately downstream of the V_H_ 8-3 region. The template as well as gRNAs targeting the V_H_ 8-3 region were transfected into WT-TET;D_H_-J_H_ RAG2-deficient pro-B cells that expressed CAS9 using electroporation. Stable clones were isolated using blasticidin as a selectable marker. Isolated clones were expanded and examined for proper insertion of the tandem MUT-TET array by PCR and a forward primer (CCTTCTAGTTGCCAGCCATCTGT) positioned in the blasticidin plasmid backbone as well as a reverse primer (GTTGGGTGAGTGTTGAATGTTCT) located immediately 3′ of second homology arm. Two clones that harbored a tandem array of MUT-TET binding sites in the V_H_ 8-3 region were identified, isolated and expanded for live-cell imaging.

### Retroviral constructs and retrovirus production

To generate Sc-Mut-TetR(B)-SNAP, mutant-TetR was generated by site directed mutagenesis of TetR-EGFP to replacing glutamic acid residues at position 37 to alanine and proline at position 39 to lysine^46^. The above-generated vector was digested by *Mfe*I and *Sal*I and ligated with a *Mfe*I-and *Sal*I-digested gBlock segment containing SG_4_ linker, Mutant-TetR, and SNAP tag to obtain Sc-Mut-TetR(B)-SNAP. Vectors were purified by CsCl gradient equilibrium centrifugation and transfected into 293T cells along with retroviral packaging vectors by calcium phosphate transfection. Medium was replaced the following morning, and viral supernatant was harvested 1 day later and stored at −80 °C until used.

### Cell culture

Pro-B cells were grown in OptiMEM media with 10% FCS and 1% Pen-Strep-Glutamine (Life Technologies) in the presence of IL7 and SCF. Supernatant from IL7-expressing cell lines was added at a concentration previously determined to promote B cell growth. Cells were incubated at 37 °C under 5% CO2. TetR-EGFP and Sc-Mut-TetR-SNAP were co-infected and after two days cells were labeled with either SNAP-cell 647-SiR or SNAP-cell TMR-Star. To promote nuclear contraction cells were treated with 10 μm STI-571 for 36 h prior to performing 3D-FISH or live-cell imaging.

### Three-dimensional (3D)-FISH

Pro-B cells were fixed in 4% paraformaldehyde for 10 min and washed in phosphate buffered saline (PBS)^49^. Cells were permeabilized for 10 min at room temperature (RT) with PBS plus 0.1% Triton X-100 plus 0.1% saponin (WB1), and followed by 20 min incubation in the presence of 20% glycerol. Coverslips were next immersed in liquid nitrogen for three freeze-thaw cycles and washed with PBS. This was followed by incubation at 37 °C in PBS, 0.1% Triton X-100 and 5% bovine serum albumin. Coverslips were next treated for 5 min in 0.1 M Tris-Cl (pH 7.2), followed by a 30 min incubation in 0.1 N HCl solution at room temperature. Coverslips were next washed once with PBS, and treated with DNase-free RNase for 1 h at 37 °C. Coverslips were washed once with PBS and incubated for 30 min in the presence of PBS, 0.5% Triton X-100 and 0.5% saponin. Coverslips were then washed with PBS followed by incubating coverslips at 73 °C in 2 × SSC, 70% formamide solution for 150 s. This was followed by a 1-min incubation in 2 × SSC, 50% formamide. Excess liquid was rapidly removed and a 10 μl of hybridization cocktail was added to each coverslip. The hybridization cocktail contained 400 ng of each labeled probe, 4 μg of mouse C_0_t DNA, 1 μg of sheared salmon-sperm DNA in 50% formamide, 2 × SSC, 10% dextran sulfate. The probes were denatured for 5 min at 70 °C and chilled on ice prior to their addition to the coverslips. BAC probes were labeled using a nick-translation kit with alexa-fluor 488-5 dUTP, alexa-fluor 568-5 dUTP or digoxigenin nick-translation kits. After overnight hybridization coverslips were removed, washed for 15 min in 2 × SSC, 50% formamide for 15 min, and three times for 5 min in 2 × SSC at 37 °C. Coverslips were mounted on slides, sealed with rubber cement, and incubated overnight at 37 °C. The V_H_ and D_H_J_H_ regions were visualized with BAC probes RP24-154H17 (mm9 coordinates: Chr12 114629838 to 114818584) and MSMG01-500B21 (mm9 coordinates: Chr12 116202300 to 116362988).

### Imaging

Live-cell imaging was performed under normal growth conditions using phenol red-free media. Cells were plated on Fluorodish poly(D-lysine) coated plates (World Precision Instruments) at least 2 h before imaging. Cells were imaged using a 100 × 1.4NA oil immersion objective on an Applied Precision OMX microscope (GE Healthcare) equipped with four EM-CCD cameras and live-cell humidified chambers for 5% CO2 supply and maintenance of temperature at 37 ℃ for both objective and stage. Twenty to thirty Z-sections were obtained with a spacing of 0.5 μm. Images were acquired at a rate of one stack every 2 s for a total of 200 total time points or one stack every 40 s for a total of 120 time points. Laser light sources of wavelengths 488 nm (for EGFP), 562 nm (for SNAP-cell TMR-STAR), or 641 nm (for SNAP-cell 647-SiR) were used. Laser intensity was 1% and exposure time was ~10–20 ms. For two-color imaging, the two colors were imaged simultaneously. All images were acquired in the conventional imaging mode of the SoftWoRx software. Data were deconvolved using Applied Precision propriety iterative-constrained deconvolution SoftWorx software. To separate signal from noise a Mexican hat filtering approach was applied using Imaris software (Bitplane). The filter size was equivalent to the size of the fluorescent signal. This was followed by extracting the x, y, and z coordinates from the intensity center of mass for each of the signals. A “track duration” filter was used to remove incorrectly recognized signals.

### Modeling the chromatin fiber as a worm-like chain

The 3 Mb Igh locus was modeled as a bead-spring self-avoiding worm-like chain, using an open source Molecular Dynamics simulation platform LAMMPS^48^. The chromatin fiber was simulated using the parameter values of 50 bp/nm for the fiber packing density and 14 nm for the bead diameter, resulting in 700 bp per bead and a total of 4298 beads in the Igh locus. These values are consistent with estimates derived from experiments^3,55^. The known genomic locations of individual V_H_, D_H_, J_H_, and CTCF elements determined the beads on which these elements were positioned in the simulations. The consecutive beads were connected by harmonic springs. The repulsive part of the Lennard-Jones potential was used to account for the physical volume occupied by each bead. The choice of parameters allowed occasional chain self-crossing, mimicking a moderate topoisomerase activity. The persistence of the chain was modeled through an angle potential, which produced a persistence length of ~22 nm. The dynamics of the chain was simulated using LAMMPS fix commands that perform Langevin dynamics according to the Langevin equation.

### Simulating a spectrum of chromatin configurations

The hierarchy of the simulated polymer models (Fig. 5a) included a simple unstructured chromatin chain, a one-loop configuration of the chain, a two-loop configuration, and a spatially confined chain mimicking the effect of an “averaged” multiple-loop configuration. The looped configurations were engineered by introducing harmonic spring-bonds between the beads that anchored the loops. The simulation of a spatially confined chain was performed by introducing a global spherical confining potential of diameter 0.8 µm to enclose the otherwise unstructured chain. To obtain the spatial distance vs genomic distance relation, a statistical ensemble of 1000 chains was generated for each chromatin configuration.

Weak intra-chain interactions were modeled by introducing 5% equidistant cross-linkable sites in the simulation of the spatially confined chain (Fig. 5b). The cross-linkable beads could undergo pairwise binding/unbinding events. The degree of reversibility of the crosslinks was varied by tuning the lifetime of the crosslinks. In order to explore the exclusive effect of crosslinks, a control set of simulations was performed by replacing the global confining potential with periodic boundary conditions.

### First-passage time analysis

A potential encounter event was identified as a dip in the distance between the V_H_ and D_H_J_H_ segments below a threshold value. The threshold value *r*_c_ was determined by combining the value of the true interaction distance *r*_int_ and the localization error *r*_err_ through *r*_c_ = (*r*_int_^2^ + *r*_err_^2^)^0.5^. The value of *r*_int_ was set to be 30 nm, and the value of *r*_err_^2^ was extracted from the control experiment on measurement-error to be 0.015 µm^2^. The time corresponding to the first dip provides an estimate for the first-passage time. The first-encounter times from experiment and simulation as a function of the mean distance within the V_H_-D_H_J_H_ pairs, as well as the distributions of the first-encounter times, were compared (Fig. 5d). The first-encounter times from experiment were fitted with a power-law relationship FPT = *β* < *r* > ^2/*α*^. The fit yielded *α* = 0.17 and *β* = 10^8^ µm^2/*α*^s. The value of *α* is close to *α* = 0.13 extracted from the fit of the MSD of STI-571 treated pro-B cells, thus validating the predicted scaling MFPT ~ *R*^2/*α*^.

### Extracting genomic motion from observed dynamics

The observed apparent relative motion of the genomic segments is a result of a superposition of the true relative motion of the genomic segments, the rotation of the cell and nucleus, and measurement-error. We developed two independent procedures to extract the true genomic motion from the observed apparent dynamics. In the first procedure, we eliminated the rotational motion by taking advantage of the availability of pairwise V_H_-D_H_J_H_ and D_H_J_H_-D_H_J_H_ spatial distances, which are unaffected by rotation, and performing the MSD analysis on the distance trajectories *r*(*t*) as MSD = < (*r*(*t*) − *r*(*t* + *τ*))^2^ > . As the changes in distances reflect the changes in the radial direction of the true relative motion in three-dimensions (3D), we refer to the MSD calculated this way as “radial MSD”. It can be shown that, under the condition |*r*(*t*) − *r*(*t* + *τ*)| ≪ *r*(*t*), the radial MSD from the observed distance trajectories, radMSD^observed^, is equal to 1/3 of the MSD of the true relative motion in 3D, MSD^genomic^, plus measurement-error, MSD^err^. The factor 1/3 reflects the fact that the radial motion represents 1 out of 3 degrees of freedom of the full 3D motion. The MSD of the genomic motion between segments in 3D is then

1 $${\mathrm{MSD}}^{{\mathrm{genomic}}} = 3\;{\mathrm{radMSD}}^{{\mathrm{observed}}} - {\mathrm{MSD}}^{{\mathrm{err}}}.$$

Equation.= (1) gives the MSD of the true relative motion for each of the two pairs of genomic segments, the intra-chromosomal V_H_-D_H_J_H_ pair and the inter-chromosomal D_H_J_H_-D_H_J_H_ pair. We assessed the contribution of the measurement-error, MSD^err^, by performing a control experiment in which a red and a green markers were inserted in the same genomic region, so that any observed relative motion between these markers would be due to measurement-error only. The MSD^err^ was calculated using the standard formula, <(***r***(*t*) − ***r***(*t* + *τ*))^2^>, where ***r***(*t*) is the displacement vector between the two markers in the control experiment. We found the value of the MSD^err^ to be ~0.02 µm^2^, or an average localization error of 22 nm for the *x*- and *y*-coordinates and 95 nm for the *z*-coordinate for each marker The MSD plots in Fig. 3 in the main text were obtained using the above procedure.

In the second procedure, we used the fact that the genomic motion can be obtained by subtracting all other contributions away from the observed motion:

2 $${\mathrm{MSD}}^{{\mathrm{genomic}}} = {\mathrm{MSD}}^{{\mathrm{observed}}} - {\mathrm{MSD}}^{{\mathrm{rotation}}} - {\mathrm{MSD}}^{{\mathrm{err}}}.$$

The separation of individual contributions is valid as long as there is no correlation among the different types of motion and measurement-error. Equation (2) applies both to the V_H_-D_H_J_H_ and D_H_J_H_-D_H_J_H_ pairs. The MSD^observed^ can be calculated directly from experimental data, MSD = < (***r***(*t*) − ***r***(*t* + *τ*))^2^ > . The MSD^err^ was assessed through the measurement-error control experiment described in the first procedure. The rotational contribution MSD^rotation^ for the V_H_-D_H_J_H_ pair was estimated as follows. By substituting Eq. (1) into Eq. (2), we found:

3 $${\mathrm{MSD}}^{{\mathrm{rotation}}} = {\mathrm{MSD}}^{{\mathrm{observed}}} - 3\;{\mathrm{radMSD}}^{{\mathrm{observed}}}.$$

As each of the terms on the right-hand side can be obtained directly from the experimental data, we used Eq. (3) to evaluate the rotational contribution MSD^rotation^ of the D_H_J_H_-D_H_J_H_ pair. We then utilized MSD^rotation^ of the D_H_J_H_-D_H_J_H_ pair to estimate MSD^rotation^ of the V_H_-D_H_J_H_ pair by noting that, on average, the amplitude of the rotational motion of a pair of segments is proportional to the mean distance between the segments in that pair:

4 $${\mathrm{MSD}}^{{\mathrm{rotation}}}\left( {{\mathrm{V}}_{\mathrm{H}} - {\mathrm{D}}_{\mathrm{H}}{\mathrm{J}}_{\mathrm{H}}} \right) \approx \left[ {\frac{{r\left( {{\mathrm{V}}_{\mathrm{H}} - {\mathrm{D}}_{\mathrm{H}}{\mathrm{J}}_{\mathrm{H}}} \right)}}{{r\left( {{\mathrm{D}}_{\mathrm{H}}{\mathrm{J}}_{\mathrm{H}} - {\mathrm{D}}_{\mathrm{H}}{\mathrm{J}}_{\mathrm{H}}} \right)}}} \right]^2{\mathrm{MSD}}^{{\mathrm{rotation}}}\left( {{\mathrm{D}}_{\mathrm{H}}{\mathrm{J}}_{\mathrm{H}} - {\mathrm{D}}_{\mathrm{H}}{\mathrm{J}}_{\mathrm{H}}} \right).$$

Here, *r*(V_H_-D_H_J_H_) and *r*(D_H_J_H_-D_H_J_H_) are the mean distances between the segments of the corresponding pairs. We then obtained the true relative genomic motion of the V_H_-D_H_J_H_ pair by substituting the MSD^rotation^ of the V_H_-D_H_J_H_ pair found from Eq. (4) into Eq. (2).

Finally, as a self-consistency check, we compared the genomic motions of the V_H_-D_H_J_H_ pair extracted via these two procedures, Eqs. (1) and (2). The two procedures yielded nearly identical results for the entire imaging time (Fig. S2), indicating that the extracted genomic motion is independent of the procedure used. Note that all the terms in Eqs. (1) and (2) were calculated from the experimental data, and therefore both approaches involve no adjustable parameters.

### Reporting summary

Further information on research design is available in the Nature Research Reporting Summary linked to this article.

## Supplementary information

Supplementary Information

Reporting Summary

Source Data

## Data Availability

All relevant data supporting the key findings of this study are available within the article and its Supplementary Information files or from the corresponding authors upon reasonable request. A source data file is provided as a Supplementary Dataset. Source data file “DNA-FISH” provides the spatial distance measurements derived from 3D-FISH as presented in Fig. 2. Source data file “Processed Data” provides the spatial distance measurements derived from live-cell imaging as presented in Fig. 3. Source data file “Raw data” provides the unprocessed spatial distance measurements and measurement-error as presented in Figs. 2 and 3. A reporting summary for this Article is available as a Supplementary Information file.

## Code availability

The code developed in this study is available upon request (yaojunz@princeton.edu).
