# Supplementary material for: Chromosome dynamics near the sol-gel phase transition dictate the timing of remote genomic interactions
Source: Nat Commun. 2019 Jun 24;10:2771. doi: 10.1038/s41467-019-10628-9 (PMC6591236; doi:10.1038/s41467-019-10628-9)
Supplement: Supplementary file 1 — Supplementary Information [file 41467_2019_10628_MOESM1_ESM.pdf]

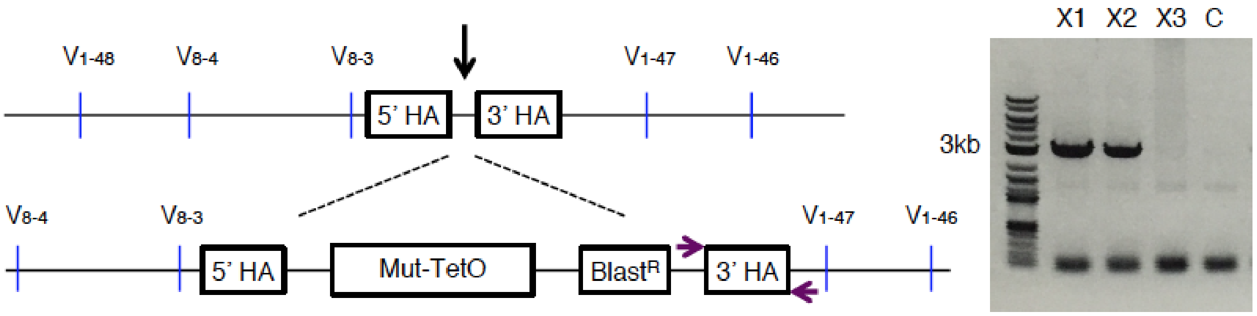

Supplementary Figure 1. Construction of template to insert MUT-TET operator binding sites into the Igh V_H region. Left panel display design of construct containing a tandem array of MUT-TET binding sites into the distal V_H region cluster. HA refers to homology arms. $Blast^R$ indicates blasticidin resistance cassette. Arrow indicated insertion of the array of MUT-TET binding sites. Primers used for genotyping proper insertions are shown. Right panel shows proper insertion of the MUT-TET operator binding sites as determined by PCR and the primers indicated in the right lower panel. V_H regions are shown on the top of blue lines.

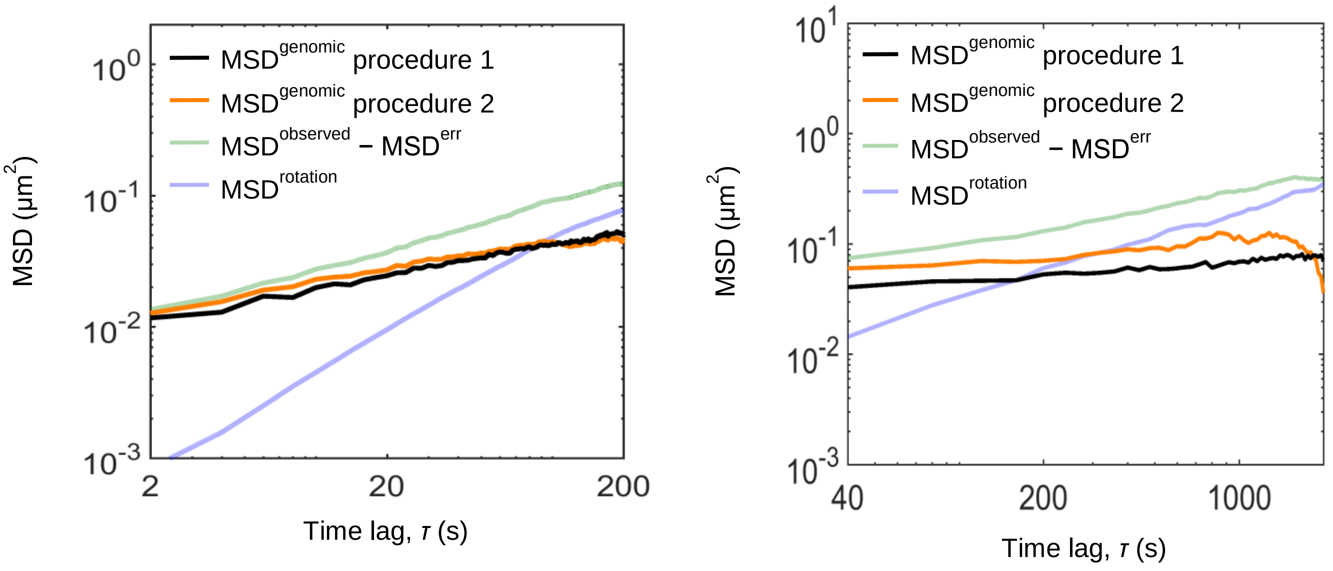

Supplementary Figure 2. Extracting V_H-D_HJ_H motion from observed motion. The relative motion of the genomic segments was extracted from the observed motion by eliminating the effect of nuclear rotation and the measurement error. To ensure that the extracted motion of the genomic segments was unaffected by the extraction procedure, two independent approaches, both involving no adjusting parameters, were utilized and demonstrated to yield nearly identical results across short (left) and long (right) time scales.

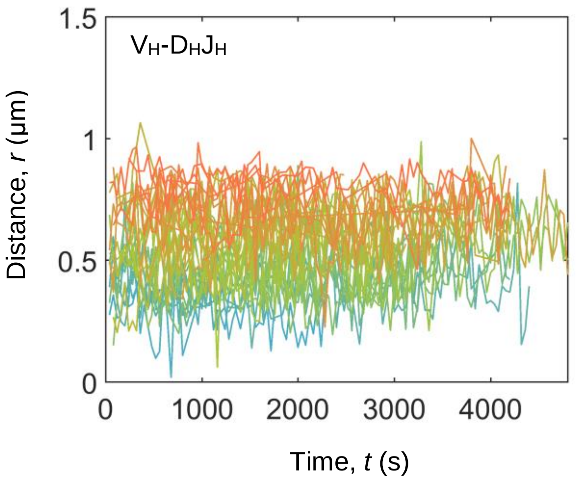

Supplementary Figure 3. Tracking V_H-D_HJ_H motion in live B cell progenitors reveals a spectrum of dynamic yet stable chromatin configurations. Temporal trajectories of V_H-D_HJ_H spatial distances, color-coded according to their mean values, reveal a de-mixing effect, visualized as the "rainbow" pattern, whereby the distances for the individual V_H-D_HJ_H pairs fluctuate around their respective mean values that remained nearly constant for at least an hour.

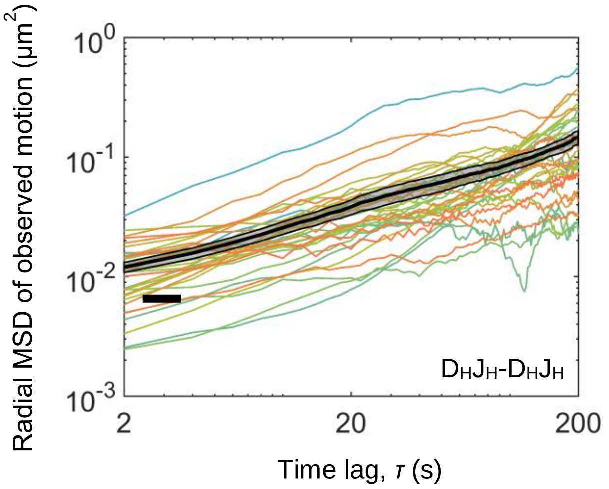

Supplementary Figure 4. Mean squared displacement of inter-chromosomal motion is subdiffusive. Time-averaged radial MSD (colored lines) and the time-and-ensemble-averaged radial MSD (black line) for the inter-chromosomal (D_HJ_H-D_HJ_H) motion in pro-B cells. Both types of MSD are subdiffusive ($\alpha < 1$) and are characterized by the similar values of the anomalous scaling exponent α (the slope) and the anomalous diffusion coefficient D (the vertical offset). The short horizontal black line indicates the mean measurement error.

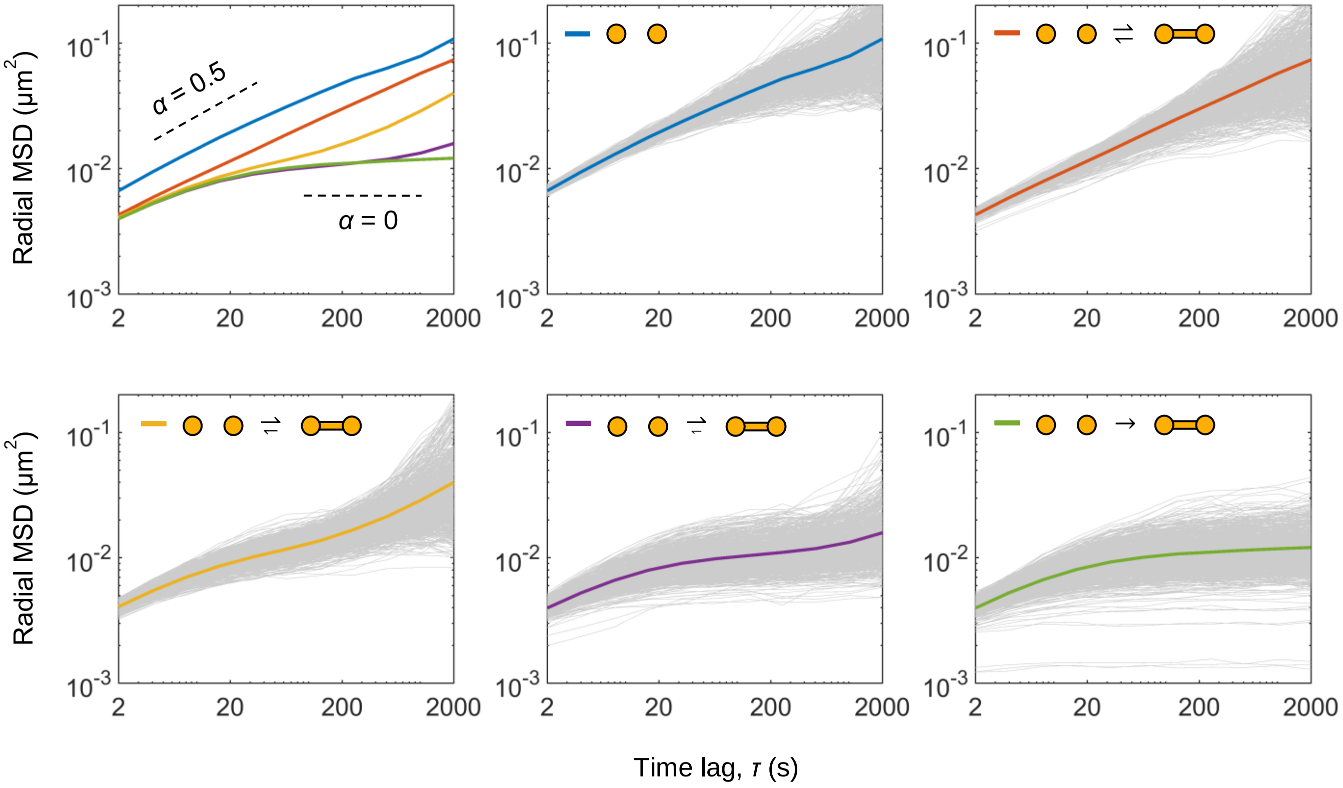

Supplementary Figure 5. The exclusive effect of crosslinks on the genomic motion. The time-averaged radial MSD (gray lines) and time-and-ensemble-averaged radial MSD (colored lines) of the relative motion between the marked V_H and D_HJ_H elements in the set of simulations with varying degree of reversibility of crosslinks and with the periodic boundary condition.

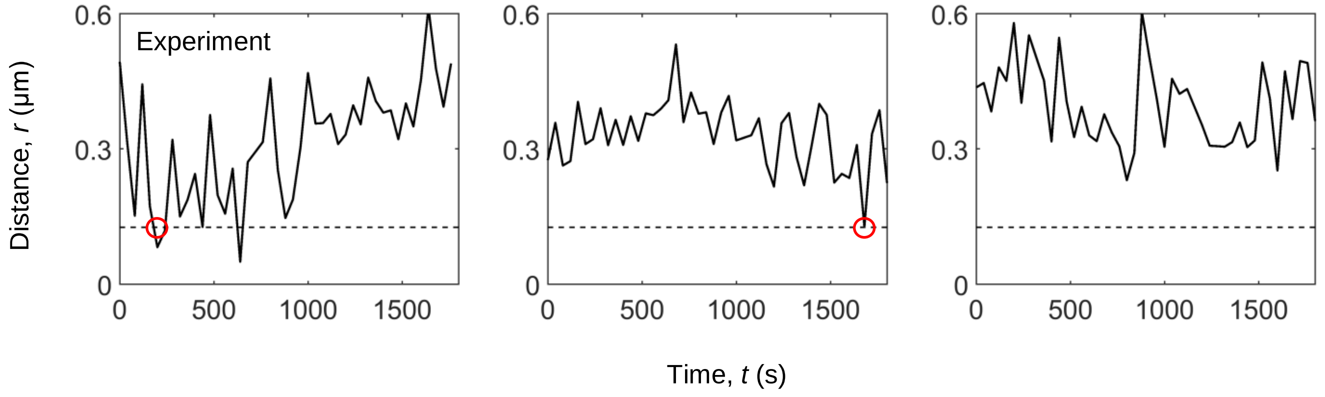

Supplementary Figure 6. Representative V_H-D_HJ_H trajectories across long time-scales. Potential first-encounter events are marked with red circles. The estimated FPTs are on the timescale of seconds to hours for the V_H and D_HJ_H elements that were found in a close spatial proximity at the start of imaging.

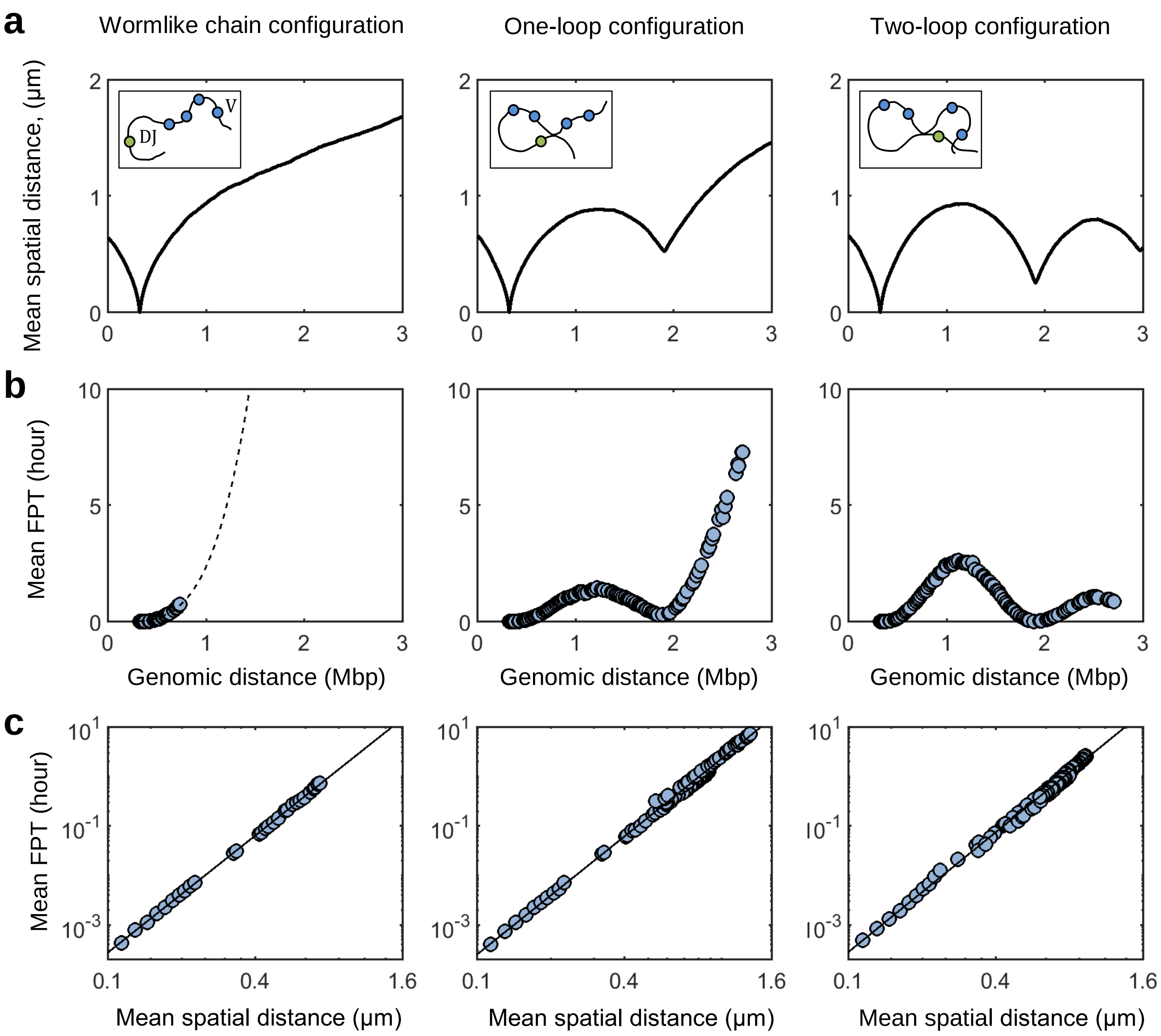

Supplementary Figure 7. Mean spatial distances and mean first-encounter times from the molecular dynamics simulations for a hierarchy of the chromatin topologies. a, Chromatin loops bring distant segments to spatial proximity. **b**, Looped configurations of chromatin reduce the mean first-encounter times between the D_HJ_H element and V_H elements from tens of hours (*left*) down to biologically relevant timescales (seconds to a few hours, *right*). **c**, Mean first-encounter times as a function of the mean V_H-D_HJ_H spatial distance for the hierarchy of chromatin topologies. The best fit results in the slope of 4, confirming the predicted scaling MFPT ~ $R^{2/\alpha}$ (α = 0.5 in the simulations).

Source of Data	Short time scale measurements		Long time scale measurements	
Type of Motion	V_H-D_HJ_H	D_HJ_H-D_HJ_H	V_H-D_HJ_H	D_HJ_H-D_HJ_H
α	0.34<br>(0.33, 0.37)	0.72<br>(0.70, 0.74)	0.21<br>(0.18, 0.24)	0.44<br>(0.41, 0.47)
D ($\mu m^2/s^\alpha$)	0.0016<br>(0.0015, 0.0016)	0.0018<br>(0.0016, 0.0019)	0.0029<br>(0.0024, 0.0034)	0.0064<br>(0.0053, 0.0075)

Supplementary Table 1. Parameter values of the scaling exponent *α* and the anomalous diffusion coefficient *D* extracted from the fit of the mean-squared displacements for V_H-D_HJ_H and D_HJ_H-D_HJ_H motion. Numbers in the parentheses denote the 95% confidence intervals of the fitted parameters.

MODELING

Extracting genomic motion from observed dynamics

The observed apparent relative motion of the genomic segments is a result of a superposition of the true relative motion of the genomic segments, the rotation of the cell and nucleus, and measurement error. We developed two independent procedures to extract the true genomic motion from the observed apparent dynamics.

In the first procedure, we eliminated the rotational motion by taking advantage of the availability of pairwise V_H-D_HJ_H and D_HJ_H-D_HJ_H spatial distances, which are unaffected by rotation, and performing the MSD analysis on the distance trajectories $r(t)$ as MSD = $\langle(r(t) - r(t + \tau))^2\rangle$. As the changes in distances reflect the changes in the radial direction of the true relative motion in three dimensions (3D), we refer to the MSD calculated this way as "radial MSD". It can be shown that, under the condition $|r(t) - r(t + \tau)| \ll r(t)$, the radial MSD from the observed distance trajectories, $\mathrm{radMSD}^{\mathrm{observed}}$, is equal to 1/3 of the MSD of the true relative motion in 3D, $\mathrm{MSD}^{\mathrm{genomic}}$, plus measurement error, $\mathrm{MSD}^{\mathrm{err}}$. The factor 1/3 reflects the fact that the radial motion represents 1 out of 3 degrees of freedom of the full 3D motion. The MSD of the genomic motion between segments in 3D is then

$$\mathrm{MSD}^{\mathrm{genomic}} = 3\,\mathrm{radMSD}^{\mathrm{observed}} - \mathrm{MSD}^{\mathrm{err}}. \qquad \text{Eq. (1)}$$

Eq. (1) gives the MSD of the true relative motion for each of the two pairs of genomic segments, the intra-chromosomal V_H-D_HJ_H pair and the inter-chromosomal D_HJ_H-D_HJ_H pair. We assessed the contribution of the measurement error, $\mathrm{MSD}^{\mathrm{err}}$, by performing a control experiment in which a red and a green markers were inserted in the same genomic region, so that any observed relative motion between these markers would be due to measurement error only. The $\mathrm{MSD}^{\mathrm{err}}$ was calculated using the standard formula, $\langle(\boldsymbol{r}(t) - \boldsymbol{r}(t + \tau))^2\rangle$, where $\boldsymbol{r}(t)$ is the displacement vector between the two markers in the control experiment. We found the value of the $\mathrm{MSD}^{\mathrm{err}}$ to be approximately 0.02 μm^2, or an average localization error of 22 nm for the *x*- and *y*-coordinates and 95 nm for the *z*-coordinate for each marker. The MSD plots in Figure 3 in the main text were obtained following the above procedure.

In the second procedure, we used the fact that the genomic motion can be obtained by subtracting all other contributions away from the observed motion:

$$\mathrm{MSD}^{\mathrm{genomic}} = \mathrm{MSD}^{\mathrm{observed}} - \mathrm{MSD}^{\mathrm{rotation}} - \mathrm{MSD}^{\mathrm{err}}. \qquad \text{Eq. (2)}$$

The separation of individual contributions is valid as long as there is no correlation among the different types of motion and measurement error. Eq. (2) applies both to the V_H-D_HJ_H and D_HJ_H-D_HJ_H pairs. The $\mathrm{MSD}^{\mathrm{observed}}$ can be calculated directly from experimental data, MSD = $<(\boldsymbol{r}(t) - \boldsymbol{r}(t + \tau))^2>$. The $\mathrm{MSD}^{\mathrm{err}}$ was assessed through the measurement error control experiment described in the first procedure. The rotational contribution $\mathrm{MSD}^{\mathrm{rotation}}$ for the V_H-D_HJ_H pair was estimated as follows. By substituting Eq. (1) into Eq. (2), we found:

$$\mathrm{MSD}^{\mathrm{rotation}} = \mathrm{MSD}^{\mathrm{observed}} - 3\,\mathrm{radMSD}^{\mathrm{observed}}. \qquad \text{Eq. (3)}$$

As each of the terms on the right-hand side can be obtained directly from the experimental data, we used Eq. (3) to evaluate the rotational contribution $\mathrm{MSD}^{\mathrm{rotation}}$ of the D_HJ_H-D_HJ_H pair. We then utilized $\mathrm{MSD}^{\mathrm{rotation}}$ of the D_HJ_H-D_HJ_H pair to estimate $\mathrm{MSD}^{\mathrm{rotation}}$ of the V_H-D_HJ_H pair by noting that, on average, the amplitude of the rotational motion of a pair of segments is proportional to the mean distance between the segments in that pair:

$$\mathrm{MSD}^{\mathrm{rotation}}(\mathrm{V_H} - \mathrm{D_HJ_H}) \approx \left[\frac{r(\mathrm{V_H}-\mathrm{D_HJ_H})}{r(\mathrm{D_HJ_H}-\mathrm{D_HJ_H})}\right]^2 \mathrm{MSD}^{\mathrm{rotation}}(\mathrm{D_HJ_H} - \mathrm{D_HJ_H}). \qquad \text{Eq. (4)}$$

Here, $r(V_H$-$D_HJ_H)$ and $r(D_HJ_H$-$D_HJ_H)$ are the mean distances between the segments of the corresponding pairs. We then obtained the true relative genomic motion of the V_H-D_HJ_H pair by substituting the $\mathrm{MSD}^{\mathrm{rotation}}$ of the V_H-D_HJ_H pair found from Eq. (4) into Eq. (2).

Finally, as a self-consistency check, we compared the genomic motions of the V_H-D_HJ_H pair extracted via these two procedures, Eq. (1) and Eq. (2). The two procedures yielded nearly identical results for the entire imaging time (Figure S2), indicating that the extracted genomic motion is independent of the procedure used. Note that all the terms in Eq. (1) and Eq. (2) were calculated from the experimental data, and therefore both approaches involve no adjustable parameters.

MOLECULAR DYNAMICS SIMULATIONS

Modeling the chromatin fiber as a worm-like chain

The mouse immunoglobulin heavy chain locus is located on chromosome 12, spanning the 3 Mb region between nucleotides 117,349,200 and 114,341,024. We modeled the Igh locus as a bead-spring self-avoiding worm-like chain, using an open source molecular dynamics simulation platform LAMMPS (Plimpton, 1995). The parameter values for the simulated chromatin fiber were chosen by combining estimates reported in multiple experiments (Jhunjhunwala et al., 2008; Sanborn et al., 2015; Ou et al., 2017). Specifically, we assumed the packing density of chromatin fiber to be 50 bp/nm and the diameter of the bead (d) to be 14 nm, leading to 700 bp per bead and a total of 4298 beads in the Igh locus. The known genomic locations of individual V_H, D_H, J_H and CTCF elements determined the beads on which these elements were positioned in the simulations. Specifically, the fluorescently marked V_H and D_HJ_H regions were positioned on beads 2712 and 467, respectively. The consecutive beads were connected through the harmonic spring potential $k(r - d)^2$ with the spring constant $k = 25k_BT/d^2$, where k_B is the Boltzmann constant and T was set to the room temperature, 300 K. To account for the physical volume occupied by each bead, we introduced repulsive interactions between all pairs of beads except neighboring beads. The repulsion was modeled using the Lennard-Jones potential, $4\varepsilon[(\sigma/r)^{12} - (\sigma/r)^6]$, with $\varepsilon = 1k_BT$ and $\sigma = d/1.12$. The potential was cut off and shifted to 0 at $r = d$ so that only the repulsive part of the potential was used in the simulation. The above choice of parameters allows occasional chain self-crossing, mimicking a moderate topoisomerase activity. The persistence of the chain was modeled through the angle potential $a\cdot\cos(\theta)$ with θ being the angle formed between three successive beads. A proper choice of the prefactor in the bending energy, $a = 2k_BT$, resulted in a persistence length of approximately 22 nm.

The dynamics of the chain was simulated using the commands fix nve and fix langevin in LAMMPS, which performs Langevin dynamics according to the Langevin equation: $m\,d^2\boldsymbol{R}_i/dt^2 = -\,\xi d\boldsymbol{R}_i/dt - dU/d\boldsymbol{R}_i + \boldsymbol{f}$. In the equation, m is the mass of the bead, $\boldsymbol{R}_i$ is the center position of the bead i, ξ is the friction coefficient, U includes all the interaction potentials described above, and $\boldsymbol{f}$ is the Gaussian-distributed

noise with $<f_\alpha(t) f_\beta(t')> = 2k_B T\xi\delta_{\alpha\beta}\delta(t - t')$, where α and β denote the *x*-, *y*-, or *z*-component of $\boldsymbol{f}$. The mass of the bead was estimated by assuming that the density of chromatin fiber is comparable to that of water. The friction coefficient ξ in the damping force term was calculated from Stokes-Einstein relation, $\xi = 3\pi\eta d$, where the dynamic viscosity η was taken to be 1 Pa·s (close to that of honey). Note that even though the reported viscosity of nucleoplasm is much lower, the crowded environment of the nucleus and non-specific adhesive interactions between molecules can result in a much higher effective viscosity for relatively large objects, such as the labeled chromatin segments. The above value of viscosity was chosen to best match the dynamical properties of segment motion observed in our experiments. To speed up the simulations, a larger value of the bead mass was used while keeping ξ (or the diffusion coefficient) constant, which led to an increased velocity relaxation time and thus allowed a larger integration time step in the simulation. This method did not affect the accuracy of the simulations as the timescale of interest was much longer than the increased velocity relaxation time, i.e. the simulations were performed in the overdamped regime.

Simulating a spectrum of chromatin configurations

The hierarchy of the simulated polymer models (Figure 4a) included a simple unstructured chromatin chain, a one-loop configuration of the chain, a two-loop configuration, and a spatially confined chain mimicking an "averaged" multiple-loop configuration. The one-loop and two-loop configurations were engineered by introducing harmonic-spring bonds between the beads that anchored the loops, using the same bond potential as the harmonic potential used for the springs connecting consecutive beads. Specifically, in the one-loop configuration, the bond was introduced between a CTCF site in the superanchor region (bead 171) and a CTCF site upstream of the marked V region (bead 2723), thus enclosing the marked V_H and $D_H J_H$ regions in the same loop. In the two-loop configuration, one bond was introduced between a CTCF site in the superanchor region (bead 171) and the most distal CTCF site (bead 4241), and another bond between the CTCF site adjacent to the marked $D_H J_H$ region (bead 550) and the CTCF site upstream of the marked V_H region (bead 2723), thus separating the marked V_H and

D_HJ_H regions in different loops. The simulation of a spatially confined chain was performed by introducing a global spherical confining potential $k(d - r)^2$ to enclose the otherwise unstructured chain. Note that r in the potential is the distance from the bead to the boundary of the sphere. The potential was only switched on when the bead was within a distance d from the boundary, $r < d$, with d=14nm. The spring constant k was set to be the same as that of the springs connecting beads. The diameter of the confining sphere was set to be 0.8 µm.

The spatial distance vs genomic distance relation was obtained from a statistical ensemble of 1000 chains for each chromatin configuration. The spatial distances between the D_HJ_H bead and the rest of beads on the chain were calculated for each chain, averaged over the ensemble, and plotted as a function of genomic distance (Figure 4a).

First-encounter times for the D_HJ_H segment and the V_H-repertoire in looped configurations

The effect of chromatin topology on the first-encounter times was examined quantitatively by performing Molecular Dynamics simulations of the hierarchy of the polymer configurations described above. Here, the packing density of chromatin fiber was set to be 130 bp/nm and the diameter of the bead to be 30 nm, leading to 772 beads in the Igh locus and a persistence length of approximately 50 nm. The marked V_H and D_HJ_H regions were positioned on beads 487 and 84. In the one-loop configuration, the bond was introduced between CTCF sites on beads 31 and 489. In the two-loop configuration, one bond was introduced between CTCF sites on beads 31 and 762, and another bond between CTCF sites on beads 99 and 489. The interaction distance, which signified a genomic encounter, was set to be 60 nm.

To find the first-encounter time, the distance between V_H and D_HJ_H beads was checked once every time interval equal to the velocity relaxation time of the beads. Once the D_HJ_H bead was within the interaction distance from a V_H bead, the corresponding V_H-D_HJ_H encounter time for that V_H segment was recorded. To speed up the simulations, a larger value of the bead mass was used. The larger value of the bead mass and the chosen frequency of checking for a potential encounter only had minor effects on the first-encounter times: a control simulation showed that, upon decreasing the bead mass (and hence

increasing the checking frequency) by a factor of 100, the first-encounter times were only reduced by a factor of 2. From the simulation, we found that the D_HJ_H segment can readily encounter V_H segments located on different loops within biologically relevant timescales (Figure S7).

Simulating crosslinks with varying degree of reversibility

The live-imaging data on the Igh locus dynamics indicated that the locus adopts a spectrum of stable configurations. To explore the possibility that weak intra-chain interactions could increase the spatial rigidity of the locus and hence stabilize the locus structure, we introduced 5% equally spaced crosslinkable sites (on beads 1, 21, 41, …) into the simulation of the spatially confined chain (Figure 4b). The crosslinkable beads could undergo pairwise binding/unbinding events. The bound beads were subjected to a harmonic interaction potential $k_b(r - d_b)^2$ with the mean bond length d_b = 50 nm and a soft spring constant, $k_b = 0.1k$. We performed a set of simulations with decreasing degree of reversibility of the crosslinks. The simulation without crosslinks (blue in Figure 5a) is the same as the original simulation of the spatially confined chain. The simulations with reversible crosslinks were performed using the commands fix bond/create and fix bond/break in LAMMPS. Bonds were checked for creation once every 0.01 s, and were created between unbound crosslinkable bead pairs when their separations were smaller than 60 nm. The created bonds were checked for breaking once every 1 s, 10 s, or 100 s (orange, yellow, purple in Figure 5a), and were broken if the separations between the bound beads were larger than 60 nm. We estimated the bond lifetimes in these simulations to be about 10 s, 100 s, and 1000 s, respectively. The simulation with irreversible crosslinks (green in Figure 5a) was performed by only using the command fix bond/create. To keep the bond information updated, we replaced the original fix_bond_create.cpp file in LAMMPS MC package, which only counts the bonds once at the beginning of the simulation run, with a modified version (Buyl and Nies, 2015). Another set of simulations, which aimed to explore the exclusive effect of crosslinks (Figure S5), was performed by replacing the global confining potential with periodic boundary conditions using a box size 0.6 µm. Each simulation in both sets of simulations with different degree of reversibility of crosslinks was repeated 10 times using a different

random seed. As V_H and D_HJ_H regions are epigenetically marked and thus may be highly interactive, their properties resemble that of the crosslinkable beads, we thus only recorded the *x*-, *y*-, and *z*-coordinates of the crosslinkable beads in each simulation run, to reduce the size of the data file. The coordinates of the crosslinkable beads in the chain were recorded once every 2 s for 4000 s to mimic the experimental protocol. To generate the V_H-D_HJ_H distance trajectories and to enrich the statistics, we have calculated the spatial distances between any two crosslinkable beads with the same genomic separation as that of the marked V_H and D_HJ_H beads (~2240 beads apart), i.e., distances between beads 1 and 2241, 21 and 2261, … were calculated at all time points. The resulting "V_H-D_HJ_H" distance trajectories were used in the MSD analysis (Figure 5a) and the first-encounter-time analysis (Figure 5d).

First-encounter-time analysis of experimental and simulated V_H-D_HJ_H trajectories

The two-color imaging strategy enabled a direct estimation of the V_H-D_HJ_H first-encounter times. A potential encounter event was identified as a dip in the distance between the V_H and D_HJ_H segments below a threshold value. The threshold value r_c was determined by combining the value of the true interaction distance r_{int} and the localization error r_{err} through $r_c = (r_{int}^2 + r_{err}^2)^{0.5}$. The value of r_{int} was set to be 30 nm, and the value of r_{err}^2 was extracted from the control experiment by averaging the squared distance between the two markers and found to be 0.015 μm^2. Note that this value for r_{err}^2 is slightly larger than the value expected theoretically, $r_{err}^2 = 0.5\ \mathrm{MSD}^{err} = 0.01$ μm^2, likely due to the effect of chromatic aberration in the experiments. As a dip below the threshold is a potential encounter event, the time corresponding to the first dip provides a lower bound for the V_H and D_HJ_H first-encounter time. On the other hand, as distance trajectories were recorded at a finite time interval, missed encounter events due to finite recording frequency may lead to an overestimation of the observed first-encounter times.

To investigate the relationship between the first-encounter times and the spatial distance, we generated a pool of contracted cells, in which the smaller spatial distances yielded richer statistics of the potential encounter events. Experimental V_H-D_HJ_H trajectories were recorded once every 2 s for 400 s. To best mimic the experimental protocol, the recorded V_H-D_HJ_H trajectories from simulations (see the

above section on how the trajectories were generated) were chopped into shorter trajectories of the duration of 400 s. Measurement errors were randomly drawn from the trajectories obtained in the control experiment and added to the simulated V_H-D_HJ_H trajectories.

The first-encounter times from experiment and simulation as a function of mean separation distance within the V_H-D_HJ_H pair, as well as the distributions of the first-encounter times, were compared (Figure 5d). The first-encounter times from experiment were fitted with a power law relationship FPT = $\beta \langle r \rangle^{2/\alpha}$. As the finite length of trajectories (400 s) sets an upper limit for the observed FPTs, the parameters α and β were found by minimizing the sum of the squares of the distance between individual data points and the fitted line along the *x*-axis. The fit yielded α = 0.17 and $\beta = 10^8$ $\mu m^{-2/\alpha}s$. The value of α is in agreement with the subdiffusive exponent α = 0.13 extracted from the fit of the MSD of contracted cells, confirming the predicted scaling MFPT ~ $R^{2/\alpha}$.

REFERENCES

Plimpton, S. (1995). Fast parallel algorithms for short-range molecular dynamics. J. Comp. Phys. 117, 1-19.

Jhunjhunwala, S., van Zelm, M.C., Peak, M.M., Cutchin, S., Riblet, R., van Dongen, J.J., Grosveld, F.G., Knoch, T.A. and Murre, C. (2008). The 3D structure of the immunoglobulin heavy-chain locus: implications for long-range genomic interactions. Cell 133, 265-279.

Sanborn, A.L., Rao, S.S.P., Huang, S.-C., Durand, N.C., Huntley, M.H., Jewett, A.I., Bochkov, I.D., Chinnappan, D., Cutkosky, A., Li, J., Geeting, K.P., Gnirke, A., Melnikov, A., McKenna, D., Stamenova, E.K., Lander, E.S., Lieberman Aiden, E. (2015). Chromatin extrusion explains key features of loop and domain formation in wild-type and engineered genomes. Proc. Natl. Acad. Sci. USA 112 (47) E6456-E6465.

Ou, H.D., Phan, S., Deerinck, T.J., Thor, A., Ellisman, M.H., O'Shea, C.C. (2017). ChromEMT: Visualizing 3D chromatin structure and compaction in interphase and mitotic cells. Science 357, 6349-6358.

de Buyl, P. and Nies, E. (2015). A parallel algorithm for step- and chain-growth polymerization in molecular dynamics. J. Chem. Phys. 142, 134102.

Lucas, J.S., Zhang, Y., Dudko, O.K. and Murre, C. (2014). 3D Trajectories adopted by coding and regulatory DNA elements: First-passage times for genomic interactions. Cell 158, 339-352.
