# Supplementary material for: Chromosome dynamics near the sol-gel phase transition dictate the timing of remote genomic interactions
Source: Nat Commun. 2019 Jun 24;10:2771. doi: 10.1038/s41467-019-10628-9 (PMC6591236; doi:10.1038/s41467-019-10628-9)
Supplement: Supplementary file 3 — Source Data [file 41467_2019_10628_MOESM3_ESM.zip › Source Data/DNAFISH/Read me DNA -FISHdocx.docx]

DNA FISH data is presented that is displayed in Figure 2b.
