# Supplementary material for: Chromosome dynamics near the sol-gel phase transition dictate the timing of remote genomic interactions
Source: Nat Commun. 2019 Jun 24;10:2771. doi: 10.1038/s41467-019-10628-9 (PMC6591236; doi:10.1038/s41467-019-10628-9)
Supplement: Supplementary file 3 — Source Data [file 41467_2019_10628_MOESM3_ESM.zip › Source Data/RawData/Readme_RawData.docx]

**Raw Data**

The raw data from live imaging measurement of pro-B cells can be found in the folder: NonContractedCells.

The raw data derived from live imaging measurement of cells in the control experiment for measurement error can be found in the folder: Measurement error.

The raw data derived from live imaging measurement of pro-B cells treated by Gleevec can be found in the folder: Contracted cells. Gleevec treatment results in nuclear and locus contraction. Data derived from contracted cells were mainly used in Figure 5b and 5d.

The Parameter folder contains: Nt.mat (total number of time steps in the measurement), Nc.mat (total number of cells), and ts.mat (step size for each time step).

**Data Format**

R1_i.txt contains the *x*, *y*, *z* coordinates of the labeled V_H_ region for cell number i, and the step number *n* at which the coordinates were recorded.

G1_i.txt contains the *x*, *y*, *z* coordinates of the labeled D_H_J_H_ region for cell number i, and the step number *n* at which the coordinates were recorded. This labeled D_H_J_H_ region is in the same allele as that of labeled V_H_ region.

G2_i.txt contains the *x*, *y*, *z* coordinates of the labeled D_H_J_H_ region for cell number i, and the step number *n* at which the coordinates were recorded. This labeled D_H_J_H_ region is in the opposite allele as that of labeled V_H_ region.

The coordinates are in unit of µm. The time at which the coordinates were recorded can be found by multiplying the step number *n* by the step size (2 s for short term imaging and 40 s for long term imaging).
