# Supplementary material for: Chromosome dynamics near the sol-gel phase transition dictate the timing of remote genomic interactions
Source: Nat Commun. 2019 Jun 24;10:2771. doi: 10.1038/s41467-019-10628-9 (PMC6591236; doi:10.1038/s41467-019-10628-9)
Supplement: Supplementary file 3 — Source Data [file 41467_2019_10628_MOESM3_ESM.zip › Source Data/ProcessedData/Readme_ProccessedData.docx]

**Processed Data**

During the measurement, the labeled V_H_ and D_H_J_H_ regions occasionally went out of the *z* stack (out of focus) or were too close to the boundary of the *z* stack, resulting om missed data points or low-quality data points. To exclude these data points, we processed the raw data for further analysis. We first synchronized the raw data using MATLAB code: DataSynchronization.m. The code selects all the time points that have the coordinates of both V_H_ and D_H_J_H_ regions on the same allele recorded, and saved them in the folder G1R1. The code selects all the time points that have the coordinates of both D_H_J_H_ regions on both alleles recorded, and saved them in the folder G1G2. The code selects all the time points that have the coordinates for all three regions recorded and saved them in the folder G1G2R1. We have also manually inspected the data, and discarded some low quality data points.

The processed data for pro-B cells can be found in the folder: NonContractedCells, and for the contracted cells can be found in the folder: ContractedCells. Note that some of the data files are empty, because all the data points were discarded after data processing, e.g., 15 cells out of 60 cells in NonContractedCells/ShortTermImaging/G1R1 were discarded. The processed data were used for data analysis in the paper.

The data format is the same as that of raw data.
